# Clinical and economic consequences of hospital-acquired resistant and multidrug-resistant *Pseudomonas aeruginosa* infections: a systematic review and meta-analysis

**DOI:** 10.1186/2047-2994-3-32

**Published:** 2014-10-20

**Authors:** Dilip Nathwani, Gowri Raman, Katherine Sulham, Meghan Gavaghan, Vandana Menon

**Affiliations:** Ninewells Hospital and Medical School, Dundee, Scotland DD19SY UK; Tufts Medical Center for Evidence Synthesis, Institute for Clinical Research and Health Policy Studies, 800 Washington Street, Box 63, Boston, MA 02111 USA; GfK Market Access, LLC, 21 Cochituate Rd, Wayland, MA 01778 USA; Cubist Pharmaceuticals, 65 Hayden Ave, Lexington, MA 02421 USA

**Keywords:** Pseudomonas aeruginosa, Resistance, All-cause mortality

## Abstract

**Background:**

Increasing rates of resistant and multidrug-resistant (MDR) *P. aeruginosa* in hospitalized patients constitute a major public health threat. We present a systematic review of the clinical and economic impact of this resistant pathogen.

**Methods:**

Studies indexed in MEDLINE and Cochrane databases between January 2000-February 2013, and reported all-cause mortality, length of stay, hospital costs, readmission, or recurrence in at least 20 hospitalized patients with laboratory confirmed resistant *P. aeruginosa* infection were included. We accepted individual study definitions of MDR, and assessed study methodological quality.

**Results:**

The most common definition of MDR was resistance to more than one agent in three or more categories of antibiotics. Twenty-three studies (7,881 patients with susceptible *P. aeruginosa*, 1,653 with resistant *P. aeruginosa*, 559 with MDR *P. aeruginosa*, 387 non-infected patients without *P. aeruginosa*) were analyzed. A random effects model meta-analysis was feasible for the endpoint of all-cause in-hospital mortality. All-cause mortality was 34% (95% confidence interval (CI) 27% – 41%) in patients with any resistant *P. aeruginosa* compared to 22% (95% CI 14% – 29%) with susceptible *P. aeruginosa.* The meta-analysis demonstrated a > 2-fold increased risk of mortality with MDR *P. aeruginosa* (relative risk (RR) 2.34, 95% CI 1.53 – 3.57) and a 24% increased risk with resistant *P. aeruginosa* (RR 1.24, 95% CI 1.11 – 1.38), compared to susceptible *P. aeruginosa*. An adjusted meta-analysis of data from seven studies demonstrated a statistically non-significant increased risk of mortality in patients with any resistant *P. aeruginosa* (adjusted RR 1.24, 95% CI 0.98 – 1.57). All three studies that reported infection-related mortality found a statistically significantly increased risk in patients with MDR *P. aeruginosa* compared to those with susceptible *P. aeruginosa.* Across studies, hospital length of stay (LOS) was higher in patients with resistant and MDR *P. aeruginosa* infections, compared to susceptible *P. aeruginosa* and control patients. Limitations included heterogeneity in MDR definition, restriction to nosocomial infections, and potential confounding in analyses.

**Conclusions:**

Hospitalized patients with resistant and MDR *P. aeruginosa* infections appear to have increased all-cause mortality and LOS. The negative clinical and economic impact of these pathogens warrants in-depth evaluation of optimal infection prevention and stewardship strategies.

**Electronic supplementary material:**

The online version of this article (doi:10.1186/2047-2994-3-32) contains supplementary material, which is available to authorized users.

## Background

*Pseudomonas aeruginosa* is a frequent causative pathogen in healthcare associated infections [1]. *P. aeruginosa* is the most common Gram-negative pathogen causing nosocomial pneumonia in the United States, and it is frequently implicated in hospital-acquired urinary tract and bloodstream infections [2–4]. In a point prevalence study conducted in Western European ICUs, *P. aeruginosa* was one of the most common organisms, constituting nearly a third (29%) of all Gram-negative isolates, and was present in 17% of all positive cultures [5]. The Infectious Disease Society of America includes *P. aeruginosa* in its list of ‘ESKAPE’ pathogens that pose the greatest public health threat due to a combination of increasing prevalence and ineffectiveness of existing antibacterial agents [6].

Rates of antibiotic resistant Gram-negative infections continue to rise worldwide, and effective therapeutic options against these infections are severely limited [7–9]. Each year in Europe, approximately 400,000 patients with hospital-acquired infections present with a resistant strain [10]. Resistance is a particular problem with *P. aeruginosa,* because of the low permeability of its cell wall [11, 12] and its ability to acquire and express multiple resistance mechanisms including porin deletions and overexpression of efflux pumps [13–17].

While the prevalence of *P. aeruginosa* in the last two decades has remained stable, the prevalence of resistant strains has increased dramatically (Table 1) [18–26]. Resistant *P. aeruginosa* infections are associated with high mortality, morbidity, and increased resource utilization and costs [27–33]. Further, the acquisition of resistance during anti-pseudomonal therapy among initially susceptible isolates and the emergence of MDR isolates make treatment even more challenging [34]. The high prevalence of resistance and resultant limited treatment options leads to inappropriate empiric therapy [24, 35], which is associated with poor clinical and economic outcomes [36–42].

**Table 1:** **Summary of data on rates of resistant** ***Pseudomonas aeruginosa***

Author (Year)	Country	Setting	Rates of resistance	Ref.
NNIS (2004)	USA	ICU	Imipenem resistance = 15%	[18]
			Quinolone resistance = 9%	
			3rd-generation cephalosporin resistance = 20%	
Obritsch *et al*. (2004)	USA	ICU	MDR *P. aeruginosa* (defined as resistance to at least three of the following four drugs: imipenem, ceftazidime, ciprofloxacin, and tobramycin) increased from 4% in 1993 to 14% in 2002	[19]
Morrow *et al*. (2013)	USA	Tertiary	Doripenem resistance = 11.4%	[20]
			Imipenem resistance = 21.9%	
			Meropenem resistance = 15.4%	
			Levofloxacin resistance = 26.0%	
			Ceftazidime resistance = 15.2%	
			Tobramycin resistance = 10.1%	
			Piperacillin / tazobactam resistance = 14.7%	
Souli et al. (2008), European Center for Disease Prevention and Control (2013)	Europe	Tertiary	Carbapenem resistance = > 25%	[21, 22]
De Francesco *et al*. (2013)	Italy	Tertiary	MDR *P. aeruginosa* (defined as resistance to 5 commonly prescribed antibiotics) increased from 2.1% in 2007 to 4.1% in 2010	[23]
Joo *et al.* (2011)	Korea	Tertiary	Ceftazidime resistance = 37%	[24]
			Piperacillin resistance = 22%	
			Imipenem resistance = 23%	
			Fluoroquinolone resistance = 24%	
			Aminoglycoside resistance = 18%	
Gales *et al*. (2001)	South America	Tertiary	MDR resistance = 8.2%	[25]
Raja *et al*. (2001)	Malaysia	Tertiary	MDR resistance = 6.9%	[26]

Several studies have examined the impact of resistant Gram-negative bacilli generally and MDR *P. aeruginosa* specifically, but there has not been an in-depth, comparative analysis of the contemporary literature reporting on mortality, morbidity and costs associated with resistant versus susceptible infection. This report is a systematic review of the clinical and economic consequences of resistant and MDR *P. aeruginosa* compared to susceptible *P. aeruginosa* and control patients without *P. aeruginosa* infections. We also conducted a meta-analysis of all-cause mortality to quantify the impact of resistant and MDR *P. aeruginosa* on this clinical outcome.

## Methods

The authors followed standard systematic review methods [43]. A systematic search was conducted in the Cochrane Library and MEDLINE. In addition, the authors manually reviewed citations from retrieved articles to ensure inclusion of all relevant literature. Appendix 1 lists the initial search strategy terms related to the pathogen (*P. aeruginosa*), mode of infection (nosocomial, hospital-acquired, healthcare-acquired, hospital-associated, healthcare-associated, and ventilator-associated), and outcomes (resource utilization including length of stay, antibiotic use/duration, procedures, inpatient costs, readmission, recurrence, and death).

Study inclusion criteria included: article published in English language; publication date between January 1, 2000 and February 28, 2013; sample size of at least 20 patients; and adult hospitalized population. Articles published before 2000 were not included to ensure that the analysis focused on contemporary literature that reflects current infection rates, resistance patterns, and clinical practice guidelines. Exclusion criteria were applied to identify special patient population subsets in which study results would not provide data applicable to the general population. Studies were not limited by the source of infection, and all infection types were included as long as resistance was present (see Table 2 for a summary of sources of infection). Unpublished gray literature was not included and no authors were directly contacted for unpublished data.

**Table 2:** **Description of studies included in systematic review**

Study (Year)	Study Design	Setting/ location	Source of Infection	Definition of Resistance / Controls	Study Population	Patients (n)	Ref.
Akhabue *et al.* (2011)	Retrospective Observational	Tertiary; USA	Genitourinary, respiratory wound, blood, tissue	Culture positive results for cefepime susceptible or resistant *P. aeruginosa*	Cefepime resistant *P. aeruginosa*	213	[61]
					Susceptible *P. aeruginosa*	2316	
Brooklyn Antibiotic Resistance Task Force (2002)	Retrospective Observational	Tertiary; USA	Genitourinary, respiratory, wound	Culture positive results for carbapenem susceptible or resistant *P. aeruginosa*	Susceptible *P. aeruginosa*	10	[60]
					Resistant *P. aeruginosa*	10	
Cao *et al.* (2004)	Retrospective Observational	Tertiary; China	Genitourinary, respiratory wound, blood, tissue	Culture positive results for susceptible or MDR *P. aeruginosa* (when the absence of susceptibility to three or more antibiotics: ceftazidime, cefepime, piperacillin, ciprofloxacin, gentamicin, and imipenem or meropenem)	MDR *P. aeruginosa*	44	[57]
					Susceptible *P. aeruginosa*	68	
Eagye *et al.* (2009)	Retrospective Observational	Tertiary; USA	Genitourinary, respiratory, wound	Culture positive results for meropenem susceptible or resistant *P. aeruginosa*; control patients were located in the same unit and on the same day as cases and without 105 DRG codes related to infection	Meropenem resistant *P. aeruginosa*	58	[53]
					Susceptible *P. aeruginosa*	125	
					Control	57	
Evans *et al.* (2007)	Retrospective Observational	Tertiary; USA	Genitourinary, respiratory, wound	Culture positive results for susceptible or resistant *P. aeruginosa*; resistance defined as resistance to all the drugs in one or more of the following antibiotic classes: aminoglycosides, cephalosporins, carbapenems, and fluoroquinolones.	Susceptible *P. aeruginosa*	73	[71]
					Resistant *P. aeruginosa*	47	
Furtado *et al.* (2009)	Retrospective Observational	ICU; Brazil	Genitourinary, respiratory, wound	Culture positive results for imipenem susceptible or resistant *P. aeruginosa*; control patients were hospitalized in the same ICU unit as the case participants and matched by time (within a 30-day interval), age (within a 10-year interval), and time at risk (control patients were imipenem susceptible)	Imipenem resistant *P. aeruginosa*	63	[62]
					Control	182	
Furtado *et al*. (2011)	Prospective Observational	Tertiary; Brazil	Respiratory	Culture positive results for imipenem susceptible or resistant *P. aeruginosa*; Isolates were screened for the presence of metallo-β-lactamases by using multiplex PCR.	São Paulo Metallo-β-lactamase (SPM-1) producing imipenem resistant *P. aeruginosa*	5	[68]
					Non-SPM-1-producing susceptible *P. aeruginosa*	24	
Gasink *et al.* (2006)	Retrospective Observational	Tertiary; USA	Respiratory, wound	Culture positive results for fluoroquinolone susceptible or resistant *P. aeruginosa*	Fluoroquinolone resistant *P. aeruginosa*	320	[64]
					Susceptible *P. aeruginosa*	527	
Hirakata *et al*. (2003)	Retrospective Observational	Tertiary; Japan	Genitourinary, respiratory, wound	Culture positive results for *bla*_IMP_-positive or negative *P. aeruginosa*; control subjects selected at random from among inpatients; control subjects samples were *bla*_IMP_-negative *P. aeruginosa*	*bla*_IMP_-positive *P. aeruginosa*	69	[69]
					*bla*_IMP_-negative *P. aeruginosa*	247	
Kaminski *et al.* (2011)	Nested case cohort	ICU; France	Respiratory	Culture positive results for Ureido/carboxypenicillin susceptible or resistant *P. aeruginosa*	Ureido/carboxypenicillin-resistant *P. aeruginosa*	70	[66]
					Susceptible *P. aeruginosa*	153	
Lambert *et al.* (2011)	Prospective Observational	ICU; EU	Respiratory	Susceptibility testing in different centers was done according to local policies	Ceftazidime resistant *P. aeruginosa*	366	[31]
					Susceptible *P. aeruginosa*	1266	
Lautenbach *et al.* (2010)	Retrospective Observational	Tertiary; USA	Genitourinary, respiratory wound, blood, tissue	Culture positive results for imipenem susceptible or resistant *P. aeruginosa*	Imipenem resistant *P. aeruginosa*	253	[63]
					Susceptible *P. aeruginosa*	2289	
Montero *et al.* (2009)	Retrospective Observational	Tertiary; Spain	Respiratory	Culture positive results for *P. aeruginosa*; MDR when the absence of susceptibility to three or more antibiotic families (betalactams, quinolones, carbapenems, and aminoglycosides); control patients also had nosocomial infection (20% with *P. aeruginosa*), but were not multidrug-resistant	MDR *P. aeruginosa*	50	[58]
					Control	50	
Morales *et al.* (2012)	Retrospective Observational	Tertiary; Spain	Genitourinary, respiratory, wound	Culture positive results for susceptible or resistant *P. aeruginosa*; MDR when strains were resistant to carbapenems, β-lactams, quinolones, tobramycin, and gentamicin	MDR *P. aeruginosa*	134	[49]
					Resistant *P. aeruginosa*	119	
					Susceptible *P. aeruginosa*	149	
Paramythiotou *et al.* (2004)	Retrospective Observational (matched)	ICU; France	Genitourinary, respiratory, wound	Culture positive results for susceptible or resistant *P. aeruginosa*; MDR were resistant to piperacillin, ceftazidime, imipenem, and ciprofloxacin; control patient was hospitalized in the same ICU as the corresponding case participant during the study period but whose microbiological cultures for *P. aeruginosa* showed no growth at any time during their ICU stay. Controls matched to cases for length of ICU stay.	MDR *P. aeruginosa*	37	[54]
					Control	34	
Peña *et al.* (2013)	Retrospective Observational	Tertiary; Spain	Respiratory	MDR defined as strains non-susceptible to > = 1 agent in > = 3 anti-pseudomonal antimicrobial categories (carbapenems, β-lactams, quinolones, tobramycin, and gentamicin)	MDR *P. aeruginosa*	27	[56]
					Susceptible *P. aeruginosa*	56	
Scheetz *et al.* (2006)	Retrospective Observational	Tertiary; USA	Blood	Culture positive results for fluoroquinolone susceptible or resistant *P. aeruginosa*	Fluoroquinolone resistant *P. aeruginosa*	79	[65]
					Fluoroquinolone susceptible *P. aeruginosa*	136	
Söderström *et al.* (2009)	Retrospective Observational (matched)	Tertiary; Finland	Wound	Culture positive results for resistant *P. aeruginosa* to ciprofloxacin, tobramycin, and a combination of piperacillin and tazobactam; A control participant with negative MDR *P. aeruginosa* culture was matched to each study participant (a drug-sensitive strain of *P. aeruginosa* was cultured from 31 control participants (48%))	MDR *P. aeruginosa*	64	[59]
					Control	64	
Tam *et al.* (2009)	Retrospective Observational	Tertiary; USA	Blood	Culture positive results for AmpC++ by ceftazidime susceptibility with and without clavulanic acid	AmpC++ *P. aeruginosa*	21	[72]
					Wild-type *P. aeruginosa*	33	
Tam *et al.* (2010)	Retrospective Observational	Tertiary; USA	Blood	MDR was defined as culture positive results for resistance to three or more of the following four classes of agents: antipseudomonal carbapenems, antipseudomonal β-lactams (penicillins and cephalosporins), aminoglycosides, and fluoroquinolones	MDR *P. aeruginosa*	25	[13]
					Susceptible *P. aeruginosa*	84	
Trouillet *et al.* (2002)	Prospective Observational	ICU; France	Respiratory	Culture positive results for piperacillin susceptible or resistant *P. aeruginosa*	Piperacillin resistant *P. aeruginosa*	34	[67]
					Piperacillin susceptible *P. aeruginosa*	101	
Tumbarello *et al.* (2013)	Retrospective Observational	ICU; Italy	Respiratory	Culture positive results for piperacillin susceptible or resistant *P. aeruginosa*; MDR if in vitro resistance to >1 antipseudomonal agent in 3 or more of the following categories: β-lactam/β-lactamase inhibitors, cephalosporins, carbapenems, quinolones and aminoglycosides	MDR *P. aeruginosa*	42	[55]
					Non-MDR *P. aeruginosa*	68	
Zavascki *et al.* (2006)	Prospective Observational	Tertiary; Brazil	Blood, genitourinary, respiratory, wound	Culture positive results for metallo-β-lactamase (MBL) –carrying versus non-MBL- carrying *P. aeruginosa*	MBL-carrying *P. aeruginosa*	86	[70]
					Non-MBL-carrying *P. aeruginosa*	212	

Two researchers with training in evidence-based methods extracted relevant data for analysis. The extracted data included study design; participant characteristics; follow-up period; method of assessing resistant *P. aeruginosa*; association between resistance status and outcome; potential confounding variables adjusted for; method of ascertaining outcome; and statistical analyses. A web-based, automated data platform (Doctor Evidence, Santa Monica, CA) further cross-calculated the data to identify any numerical discrepancies (i.e., mismatches of sub-data to main population data, data reported in percentiles conflicting with unit data and vice versa). A third evidence-based review produced the final digital data repository. The web-based platform assessed identified studies for inclusion. Meta-analysis was conducted using the random effects method of weighting data for pooling [44]. The results are reported as summary relative risk (RR).

We considered the following outcomes for inclusion in a meta-analysis: all-cause mortality in hospital, length of stay (hospital and ICU), hospital costs or charges, microbiological infection clearance and readmissions. The random effects meta-analyses assessed any potential differential impact of resistant and MDR pathogens on the outcomes of interest using unadjusted and adjusted data, when available. All presented p-values were obtained from analysis within included studies. The Cochrane Q Chi-square test was used to test for heterogeneity of results across studies and quantified with I^2^ [45]. In addition, we combined studies using logit transformation of individual proportions and calculated their confidence interval (CI) and then back transformed the combined value. For meta-analyses with at least 10 studies, we evaluated the potential for publication bias with funnel plots and Egger’s tests for small study effects [46]. We looked for differences across studies using stratified analyses to explain heterogeneity in association results. To assess study quality, we applied quality questions from the Newcastle-Ottawa Quality Assessment Scales for case–control and observational studies [47]. When feasible, sensitivity analyses were conducted by excluding studies of high risk of bias. All analyses were performed in Stata version 13 (StataCorp, College Station, Texas).

Studies that used hospital costs or charges as a defined primary or secondary endpoint were included in this analysis. Hospital costs and charges were inflated to 2012 US dollars using the Consumer Price Index (CPI) as reported by the Bureau of Labor Statistics [48]. In one study where the costs were reported in Euros [49], conversion to USD was performed according the exchange rate at the time of the original analysis.

This review was based on evaluation of data from published studies and was exempt from ethics committee approval. In addition, this review did not involve any direct research on patients, and no informed consent was required.

## Results and discussion

### Study characteristics

**Figure 1:**
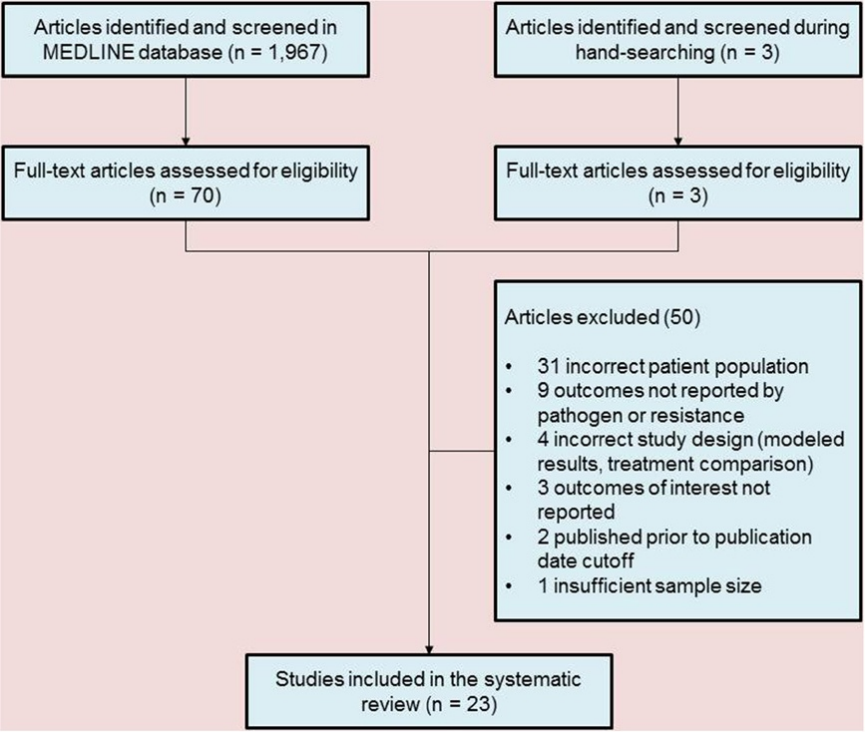
**Literature review study flow diagram.**

Our literature search identified 1,970 potentially relevant references (1,967 references and three full-text articles identified through manual searching), which contained terms from the search strategy listed (Appendix 1). Based on review of abstracts only, 73 full text articles met the necessary inclusion criteria previously mentioned. After review of full text, an additional 50 articles were excluded based on the same criteria (Figure 1).

Table 2 summarizes the definitions of controls, susceptibility, resistance, and MDR used in the studies included in the systematic review. Of note, consistent with other reports, we found that the definition of resistance and MDR in *P. aeruginosa* infections was inconsistent [50–52]. Resistance was either based on a class of antimicrobials or to a specific agent. Similarly, the most common definition for MDR was laboratory-confirmed resistance to more than one agent in three or more of categories of antibiotics, though one study required evidence of resistance to five different agents [49].

Of the 23 studies that met inclusion criteria, two were prospective observational studies, 18 were retrospective (Table 2). Of the 20 studies reporting on hospitalized patients, six specifically studied ICU patients. The 23 studies represented a total of 10,570 patients, of which 7,881 had susceptible *P. aeruginosa* infections, 1,653 had resistant *P. aeruginosa* infections, 559 had MDR *P. aeruginosa* infections, and 387 were control patients defined as non-infected patients without *P. aeruginosa* infections. Of the 23 studies, 2 were rated as good quality (low risk of bias), 17 were of moderate quality (medium risk of bias) and 4 were of poor quality (high risk of bias) (Additional file 1). Most studies included a mixture of patients with genitourinary, respiratory, wound, and bloodstream as the source of infection.

The most common definition (43.5% of studies) for MDR was laboratory-confirmed resistance to more than one agent in three or more of categories of antibiotics [13, 17, 49, 53–59]. Resistance (non-MDR) was defined as laboratory confirmed resistance to one particular antibiotic agent. Analysis of resistance included one study on carbapenem-resistant *P. aeruginosa* (n = 10) [60], one study on cefepime-resistant *P. aeruginosa* (n = 213) [61], one study on meropenem-resistant *P. aeruginosa* (n = 58) [53], two studies on imipenem-resistant *P. aeruginosa* (n = 316) [62, 63], two studies on fluoroquinolone-resistant *P. aeruginosa* (n = 399) [64, 65], one study on ceftazidime-resistant *P. aeruginosa* (n = 366) [31], one study on ureido/carboxypenicillin-resistant *P. aeruginosa* (n = 70) [66] and one study on piperacillin-resistant *P. aeruginosa* (n = 34) [67] and three studies on metallo-β-lactamase-carrying *P. aeruginosa* (MBL-PA)[68–70]. Three studies included resistant *P. aeruginosa* patients but did not define resistance specific to any one treatment (n = 187) [49, 71, 72].

Three studies reported long-term clinical outcomes in patients with MDR *P. aeruginosa*, but did not provide data regarding in-hospital outcomes and therefore, were excluded from meta-analysis [58, 59, 72].

Of the seven outcomes examined, in-hospital all-cause mortality was the only endpoint for which a meta-analysis was feasible; when only ICU mortality was reported, those data were included in the analysis of in-hospital mortality. For the other six outcomes, a meta-analysis was not possible due to insufficient data or heterogeneity of data. For the all-cause mortality analysis, we were unable to stratify by the type of infection owing to the small sample size.

### Mortality outcomes

Twenty studies reported data on hospital all-cause mortality (Table 3). In-hospital all-cause mortality ranged from 25 to 60% in the MDR *P. aeruginosa* group, 15 to 59% in the resistant *P. aeruginosa* group, and 7 to 50% in the susceptible *P. aeruginosa* group. Mortality was 34% (95% confidence interval (CI) 27% – 41%) in patients with resistant and MDR *P. aeruginosa* compared to 22% (95% CI 14% – 29%) with susceptible *P. aeruginosa.* When comparing patients with resistant *P. aeruginosa* infections versus those with susceptible *P. aeruginosa* infections, resistance was associated with 24% higher risk of in-hospital all-cause mortality (11 studies, 9,082 participants unadjusted RR 1.24, 95% CI 1.11 – 1.38; *I*^*2*^ 24.6%). Comparing patients with MDR *P. aeruginosa* infections with patients with susceptible *P. aeruginosa* infections (5 studies, 697 participants), the MDR *P. aeruginosa* group had a greater than 2-fold increase in risk of in-hospital all-cause mortality (unadjusted RR 2.34, 95% CI 1.53 – 3.57; *I*^*2*^ 78.8%).

**Table 3:** **Studies describing in-hospital mortality in patients with resistant and multidrug-resistant** ***Pseudomonas aeruginosa*** **infections**

Setting	Author	Study groups (n)	In-Hospital Mortality	Reported P-value	Ref
Tertiary care center	Akhabue *et al.* (2011)	Cefepime resistant *P. aeruginosa* (213)	20%	0.007	[61]
		Susceptible *P. aeruginosa* (2316)	13%		
	Brooklyn Antibiotic Resistance Task Force (2002)	Carbapenem resistant *P. aeruginosa* (10)	20%	> 0.05 (NS)	[60]
		Susceptible *P. aeruginosa* (10)	10%		
	Cao *et al.* (2004)	MDR *P. aeruginosa* ^d^ (44)	55%	0.05	[57]
		Susceptible *P. aeruginosa* (68)	16%		
	Eagye *et al.* (2009)	Meropenem resistant *P. aeruginosa* (58)	31%	0.152^a^	[53]
		Meropenem susceptible *P. aeruginosa* (125)	15%		
		Meropenem resistant *P. aeruginosa* (58)	31%	0.01^a^	
		Control (57)	9%		
	Evans *et al.* (2007)	Resistant *P. aeruginosa* ^e^ (47)	15%	0.43	[71]
		Susceptible *P. aeruginosa* (73)	21%		
	Furtado *et al*. (2011)	SPM-1-producing imipenem resistant *P. aeruginosa* (5)	60%	0.59	[68]
		Non-SPM-1-producing susceptible *P. aeruginosa* (24)	75%		
	Gasink *et al.* (2006)	Fluoroquinolone resistant *P. aeruginosa* (320)	24%	0.004	[64]
		Fluoroquinolone susceptible *P. aeruginosa* (527)	16%		
	Hirakata *et al*. (2003)	*bla*_IMP_-positive *P. aeruginosa* (69)	30.4%	0.41	[69]
		*bla*_IMP_-negative *P. aeruginosa* (247)	25.5%		
	Lautenbach *et al.* (2010)	Imipenem resistant *P. aeruginosa* (253)	17%	0.01	[63]
		Imipenem susceptible *P. aeruginosa* (2289)	13%		
	Morales *et al.* (2012)	MDR *P. aeruginosa* ^f^ (134)	25%	< 0.05	[49]
		Susceptible *P. aeruginosa* (149)	13%		
		Resistant *P. aeruginosa* (119)	22%	<0.05	
		Susceptible *P. aeruginosa* (149)	13%		
	Peña *et al.* (2013)	Non-MDR *P. aeruginosa* (27)	55%	0.33	[56]
		MDR *P. aeruginosa* ^g^ (56)	50%		
	Scheetz *et al.* (2006)	Fluoroquinolone resistant *P. aeruginosa* (79)	32%	0.731	[65]
		Fluoroquinolone susceptible *P. aeruginosa* (136)	29%		
	Tam *et al.* (2010)	MDR *P. aeruginosa* ^h^ (25)	56%	0.001	[13]
		Susceptible *P. aeruginosa* (84)	17%		
	Zavascki *et al*. (2006)	MBL-carrying *P. aeruginosa* (86)	51.2%	0.003	[70]
		Non-MBL-carrying *P. aeruginosa* (212)	32.1%		
ICU	Lambert *et al.* (2011)	Ceftazidime resistant *P. aeruginosa* (362)^b^	43%	NR	[31]
		Susceptible *P. aeruginosa* (1251)^b^	37%		
		Ceftazidime resistant *P. aeruginosa* (82)^c^	41%		
		Susceptible *P. aeruginosa* (280)^c^	39%		
	Furtado *et al.* (2009)	Imipenem resistant *P. aeruginosa* (63)	49%	0.02	[62]
		Control (182)	34%		
	Kaminski *et al.* (2011)	Ureido/carboxypenicillin resistant *P. aeruginosa* (70)	43%	0.56	[66]
		Ureido/carboxypenicillin susceptible *P. aeruginosa* (153)	44%		
	Paramythiotou *et al.* (2004)	MDR *P. aeruginosa* ^i^ (34)	47%	0.8	[54]
		Control (34)	50%		
	Trouillet *et al.* (2002)	Piperacillin resistant *P. aeruginosa* (34)	59%	> 0.05 (NS)	[67]
		Piperacillin susceptible *P. aeruginosa* (101)	50%		
	Tumbarello *et al*. 2013	MDR *P. aeruginosa* ^j^ (42)	60%	0.01	[55]
		Susceptible *P. aeruginosa* (68)	35%		

^a^Adjusted for time at risk; ^b^Patients with pneumonia; ^c^Patients with bloodstream infections; ^d^Absence of susceptibility to three or more antibiotics: ceftazidime, cefepime, piperacillin, ciprofloxacin, gentamicin, and imipenem or meropenem; ^e^Absence of susceptibility to all the drugs in one or more of the following antibiotic classes: aminoglycosides, cephalosporins, carbapenems, and fluoroquinolones; ^f^Absence of susceptibility to carbapenems, β-lactams, quinolones, tobramycin, and gentamicin; ^g^Absence of susceptibility to one or more agent in three or more anti-pseudomonal antimicrobial categories (carbapenems, β-lactams, quinolones, tobramycin, and gentamicin); ^h^Absence of susceptibility to three or more of the following four classes of agents: antipseudomonal carbapenems, antipseudomonal β-lactams (penicillins and cephalosporins), aminoglycosides, and fluoroquinolones; ^i^Absence of susceptibility to piperacillin, ceftazidime, imipenem, and ciprofloxacin; ^j^Absence of susceptibility to one or more antipseudomonal agent in 3 or more of the following categories: β-lactam/β-lactamase inhibitors, cephalosporins, carbapenems, quinolones and aminoglycosides.

**Figure 2:**
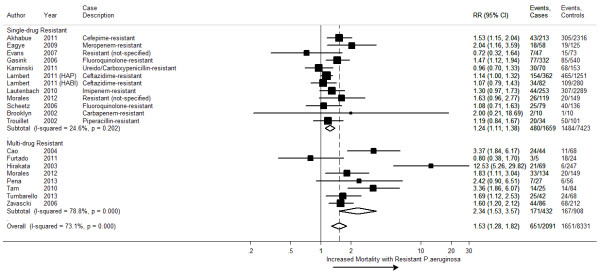
**A forest plot of unadjusted in-hospital all-cause mortality comparing resistant and susceptible** ***P. aeruginosa.*** HAP =  Health-acquired Pneumonia. HABI = Health-acquired Blood Infection.

We conducted a meta-analysis of 18 studies containing 20 comparisons (10,422 participants) that reported in-hospital all-cause mortality in patients with any resistant *P. aeruginosa* versus susceptible *P. aeruginosa* infections. The two additional comparisons came from one study that contributed data for both resistant and MDR *P. aeruginosa* groups [49] and another study that contained two different infection-site comparisons [63]. Using data reported from 18 studies, the meta-analysis demonstrated an increased risk of mortality with any resistant *P. aeruginosa* (unadjusted (RR) 1.53, 95% CI 1.28 – 1.82, *I*^*2*^ 73.1%), compared to susceptible *P. aeruginosa* (Figure 2).

**Figure 3:**
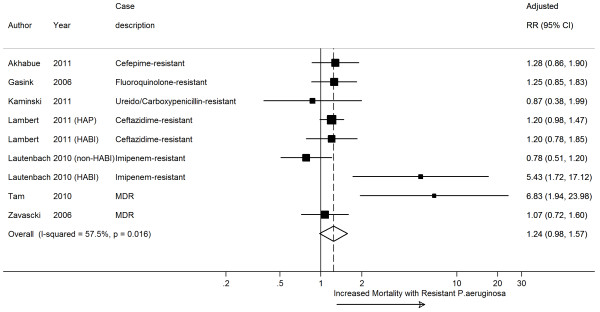
**A forest plot of adjusted in-hospital all-cause mortality comparing resistant and susceptible** ***P. aeruginosa.*** HAP = Health-acquired Pneumonia. HABI = Health-acquired Blood Infection.

Only seven studies reported data for an adjusted meta-analysis (Additional file 2: Table S1); adjusted data from these studies demonstrated an increased risk of mortality (adjusted RR 1.24, 95% CI 0.98 – 1.57, *I*^*2*^ 57.5%) that did not reach statistical significance probably due to the small sample size (Figure 3). We were unable to perform comparisons against control patients without *P. aeruginosa* infections due to a scarcity of studies reporting mortality end points in this group of patients [55, 62]).

For both unadjusted and adjusted data, stratified meta-analyses by study-level characteristics identified studies with prospective follow-up or those conducted at multi-center or ICU as less heterogeneous (Additional file 2: Table S2-S3); however, excluding high risk of bias studies did not change the overall estimate or measures of consistency (*I*^*2*^).

**Figure 4:**
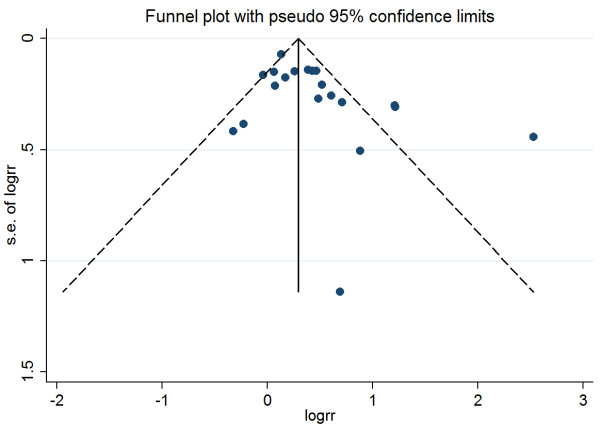
**Funnel plot with pseudo 95% confidence limits.**

Funnel plots of all studies reporting unadjusted mortality (Figure 4) indicate a potential for missing studies with inverse associations (RR <1.0). Since <10 studies reported adjusted mortality data, we did not assess publication bias for this analysis.

Across three studies that reported infection-related mortality, all found a statistically significantly increased risk in patients with MDR *P. aeruginosa* compared to those with susceptible *P. aeruginosa* (Table 4)*.*

**Table 4:** **Studies describing in-hospital mortality in patients with resistant and multidrug-resistant** ***Pseudomonas aeruginosa*** **infections – cause of death identified specifically as** ***Pseudomonas aeruginosa*** **infection**

Setting	Author	Study groups (n)	Infection-related In-Hospital Mortality	P-value	Ref
Tertiary Care Center	Cao *et al*	MDR *P. aeruginosa* ^a^ (44)	45.5%	<0.05	[57]
		Susceptible *P. aeruginosa* (68)	7.4%		
	Hirakata *et al*	*bla*_IMP_-positive *P. aeruginosa* (69)	5.8%	0.02	[69]
		*bla*_IMP_-negative *P. aeruginosa* (247)	1.2%		
	Tam *et al*	MDR *P. aeruginosa* ^b^ (25)	52%	<0.001	[13]
		Susceptible *P. aeruginosa* (84)	9.5%		

^a^Absence of susceptibility to three or more antibiotics: ceftazidime, cefepime, piperacillin, ciprofloxacin, gentamicin, and imipenem or meropenem; ^b^Absence of susceptibility to three or more of the following four classes of agents: antipseudomonal carbapenems, antipseudomonal β-lactams (penicillins and cephalosporins), aminoglycosides, and fluoroquinolones.

### Economic outcomes

Ten studies containing 12 comparisons reported hospital length of stay (Table 5). Definitions and measures for length of stay varied across eligible studies making direct comparisons challenging. The study by Paramythiotou *et. al.* was a case control study that matched MDR *P. aeruginosa* cases and controls (defined as patients without *P. aeruginosa* infections) on the basis of median length of stay in the ICU (45 versus 44 days). Therefore, we did not include these control patients in analyses of comparative ICU length of stay. Of note, this study reported hospital stay following ICU discharge of 11.1 days (standard deviation [SD] of 18.6) in the MDR *P. aeruginosa* group and 5.8 days (SD 10.4) in the control group (p = 0.28) [54].

**Table 5:** **Studies describing hospital length of stay in patients with resistant and multidrug-resistant** ***Pseudomonas aeruginosa*** **infections**

Author	Study groups (n)	Median (mean) Hospital Length of Stay (days)	Reported Significance (p-value or 95% CI)	Ref
		Main findings		
Brooklyn Antibiotic Resistance Task Force (2002)^*^	Carbapenem susceptible *P. aeruginosa* (10)	20 (17)	0.002 (0.001)	[60]
	Carbapenem resistant *P. aeruginosa* (10)	33.5 (36)		
Eagye *et al.* (2009)^*^	Meropenem resistant *P. aeruginosa* (58)	30	<0.001	[53]
	Meropenem susceptible *P. aeruginosa* (125)	16		
	Meropenem resistant *P. aeruginosa* (58)	30	<0.001	
	Control (57)	10		
Evans *et al.* (2007)^‡^	Susceptible *P. aeruginosa* (73)	20	0.03	[71]
	Resistant *P. aeruginosa* ^e^ (47)	26		
Furtado *et al.* (2009)^‡^	Imipenem resistant *P. aeruginosa* (63)	25	0.006	[62]
	Control (182)	15		
Gasink *et al.* (2006)^*^	Fluoroquinolone resistant *P. aeruginosa* (320)	10	0.13	[64]
	Fluoroquinolone susceptible *P. aeruginosa* (527)	9		
Kaminski *et al.* (2011)^†^	Ureido/carboxypenicillin resistant *P. aeruginosa* (70)	50	0.24	[66]
	Ureido/carboxypenicillin susceptible *P. aeruginosa* (153)	47.5		
Lautenbach *et al.* (2010)^†^	Imipenem resistant *P. aeruginosa* (253)	16	<0.001	[63]
	Imipenem susceptible *P. aeruginosa* (2289)	9		
Morales *et al.* (2012)^‡^	MDR *P. aeruginosa* ^f^ (134)	39 (45.7)	<0.001^a^	[49]
	Susceptible *P. aeruginosa* (149)	20 (25.1)		
	Resistant *P. aeruginosa* (119)	30 (39.0)	<0.001^a^	
	Susceptible *P. aeruginosa* (149)	20 (25.1)		
Paramythiotou *et al.* (2004)^†^	MDR *P. aeruginosa* ^g^ (34)	44.5 (57.0)^b,d^/11.1^c,d^	0.28/0.81	[54]
	Control (34)	43.5 (46.9)^b,d^/5.8^c,d^		
Tam *et al.* (2010)^†^	MDR *P. aeruginosa* ^h^ (25)	26.4^c^	0.12	[13]
	Susceptible *P. aeruginosa* (84)	16.5^c^		

^*^Length of stay reported after identification of infection with *P. aeruginosa;* ^†^Length of stay reported as total length of stay; ^‡^Length of stay not defined as total length of stay or length of stay after identification of infection with *P. aeruginosa*; ^a^ P-value reported for mean length of stay; ^b^ Length of ICU stay (ICU stay matched between cases and controls); ^c^Length of stay in hospital following ICU discharge; ^d^Study reported mean length of stay; ^e^Absence of susceptibility to all the drugs in one or more of the following antibiotic classes: aminoglycosides, cephalosporins, carbapenems, and fluoroquinolones; ^f^Absence of susceptibility to carbapenems, β-lactams, quinolones, tobramycin, and gentamicin; ^g^ Absence of susceptibility to piperacillin, ceftazidime, imipenem, and ciprofloxacin; ^h^Absence of susceptibility to three or more of the following four classes of agents: antipseudomonal carbapenems, antipseudomonal β-lactams (penicillins and cephalosporins), aminoglycosides, and fluoroquinolones.

Two studies reported mean hospital length of stay among patients with MDR *P. aeruginosa*; one study reported total length of stay (46 days, SD 29) [49] and the other reported mean length of hospital stay associated with bacteremia (26.4 days; SD 28.3) [13]. Among patients with resistant *P. aeruginosa*, 8 studies reported median hospital length of stay in patients with bacteremia ranging from 10 to 50 days [13, 49, 53, 54, 60, 62–64, 66, 71]; and 6 studies reported median hospital length of stay in susceptible *P. aeruginosa* infections ranging from 9 to 48 days [13, 49, 53, 60, 63, 64, 66, 71]. Two studies reported median hospital length of stay in control patients (10 and 15 days, respectively) [53, 54, 62].

Three studies examined length of stay in the ICU (Table 6). One study reported mean ICU length of stay of 47 days in patients with MDR *P. aeruginosa* [54]. As described previously, the control group in the analysis by Paramythiotou *et. al.* [54] matched control patients with cases on the basis of ICU length of stay and therefore we did not include this control group. Two studies reported median ICU length of stay of 13 days (interquartile range 2–36) versus 6 days (interquartile range 0–16) in one study [66, 71] and was similar in both groups in the other study (29 days) [66, 71].

**Table 6:** **Studies describing intensive care unit length of stay in patients with resistant and multidrug-resistant** ***Pseudomonas aeruginosa*** **infections**

Author	Study groups (n)	Median ICU Length of Stay (days)	Reported Significance (p-value or 95% CI)	Ref
		Main findings		
Evans *et al.* (2007)	Susceptible *P. aeruginosa* (73)	6	0.02	[71]
	Resistant *P. aeruginosa* ^c^ (47)	13		
Kaminski *et al.* (2011)	Ureido/carboxypenicillin resistant *P. aeruginosa* (70)	29	0.37	[66]
	Ureido/carboxypenicillin susceptible *P. aeruginosa* (153)	28.5		
Paramythiotou *et al.* (2004)	MDR *P. aeruginosa* ^d^ (34)	44.5 (57.0)^a,b^	0.55	[54]
	Control (34)	43.5 (46.9)^a,b^		

^a^ Length of ICU stay (ICU stay matched between cases and controls); ^b^ Study reported mean length of stay; ^c^ Absence of susceptibility to all the drugs in one or more of the following antibiotic classes: aminoglycosides, cephalosporins, carbapenems, and fluoroquinolones; ^d^ Absence of susceptibility to piperacillin, ceftazidime, imipenem, and ciprofloxacin.

Five studies reported outcomes related to hospital costs (Table 7). Four of five studies reported inpatient care costs. Of the four studies that reported inpatient care costs, two [49, 63] reported only those costs incurred after microbiological infection confirmation. One study reported hospital charges incurred after microbiological infection confirmation [64]. Additionally, one study reported costs from a hospital in Spain [49] and therefore cannot be directly compared to the remaining three studies that represent US hospital setting costs. Studies also varied in the reporting of mean versus median costs. For the two US studies that reported median costs, costs were higher in the resistant *P. aeruginosa* groups (median = $99,672, interquartile range (IQR) = $43,714 - $187,260 [71]; median = $100,704, IQR = $27,710 - $183,125 [53]) compared to patients with susceptible *P. aeruginosa* infections (median = $69,502, IQR = $24,853 - $113,933 [71]; median = $32,594, IQR = $13,112 - $100,702 [53]) and control patients (median = $25,744, IQR = $17,456 - $40,616 [71]).

**Table 7:** **Studies describing hospital costs or charges in patients with resistant and multidrug-resistant** ***Pseudomonas aeruginosa*** **infections**

Author	Study groups (n)	Description of cost	Hospital Inpatient Care Costs or Charges	Reported P-value	Ref
			Main findings		
Eagye *et al.* (2009)	Meropenem resistant *P. aeruginosa* (58)	Median total cost (IQR)	$100,704 ($27,710 – $183,125)	< 0.001	[53]
	Meropenem susceptible *P. aeruginosa* (125)		$32,594 ($13,112 – $100,702)		
	Meropenem resistant *P. aeruginosa* (58)		$100,704 ($27,710 – $183,125)	< 0.001	
	Control (57)		$25,744 ($17,456 – $40,616)		
Evans *et al.* (2007)	Susceptible *P. aeruginosa* (73)	Median total cost (IQR)	$99,672 ($43,714 – $187,260	0.015	[71]
	Resistant *P. aeruginosa* ^a^ (47)		$69,502 ($24,853 – $113,933)		
Gasink *et al.* (2006)	Fluoroquinolone resistant *P. aeruginosa* (320)	Median total charges (IQR)^*^	$62,325 ($22,129 - $188,979)	0.008	[64]
	Fluoroquinolone susceptible *P. aeruginosa* (527)		$48,733 ($18,760 - $124,820)		
Lautenbach *et al.* (2010)	Imipenem resistant *P. aeruginosa* (253)	Mean hospital cost after culture sampling (95% CI)	$286,417 ($234,326 – $338,510)	< 0.001	[63]
	Imipenem susceptible *P. aeruginosa* (2289)		$189,274 ($172,428 – $206,121)		
Morales *et al.* (2012)	MDR *P. aeruginosa* ^b^ (134)	Mean hospital cost after culture sampling (median)	$13,178 ($5,745)	< 0.001^†^	[49]
	Susceptible *P. aeruginosa* (149)		$4,258 ($2,420)		
	Resistant *P. aeruginosa* (119)		$10,662 ($5,248)	< 0.001^†^	
	Susceptible *P. aeruginosa* (149)		$4,258 ($2,420)		

^*^Hospital charges reported after identification of infection with P. aeruginosa; ^†^P-value reported for mean length of stay; ^a^Absence of susceptibility to all the drugs in one or more of the following antibiotic classes: aminoglycosides, cephalosporins, carbapenems, and fluoroquinolones; ^b^Absence of susceptibility to carbapenems, β-lactams, quinolones, tobramycin, and gentamicin.

### Other outcomes

Need for mechanical ventilation was studied as an index of patient outcomes in one case control study; patients with MDR *P. aeruginosa* required more days of mechanical ventilation than patients with susceptible *P. aeruginosa* (15 versus 11 days) [55]. One study reported persistence of microbiological infection of 75% and clinical persistence or recurrence of 38% in patients with MDR *P. aeruginosa* infection versus 61% and 39% respectively, in the non-MDR *P. aeruginosa* group [56]. As noted in the Methods section, meta-analyses were not performed for these outcomes due to insufficient data and heterogeneity of the literature.

## Conclusions

This systematic review of the literature identified some strengths and weaknesses in relation to published data on the impact of *P. aeruginosa* infections on clinical and economic outcomes. The meta-analysis of studies examining the impact of resistance or MDR infection indicates a significant increase in hospital all-cause mortality compared to infections due to susceptible pathogens. Patients with resistant and MDR *P. aeruginosa* infections versus those with susceptible *P. aeruginosa* infections had a relative risk of 1.24 and 2.31, respectively.

In keeping with previous literature [27, 28, 32], our review suggests that MDR and resistant *P. aeruginosa* infections are associated with higher hospital and ICU length of stay as an outcome compared to susceptible *P. aeruginosa* infections; however, there was a scarcity of studies examining length of stay in patients with resistant *P. aeruginosa* infections compared to control patients without infection. Further research is needed into the economic impact of resistant *P. aeruginosa* infections and future studies must take into account regional and national differences in standards of care, and other critical patient level factors including community versus hospital acquired infection, duration of hospitalization prior to the infection, severity of illness, the timeliness and appropriateness of therapy, time at risk of infection, and site of infection [73].

Among reviewed studies, only one study highlighted inadequate initial antibiotic therapy as a significant risk factor for in-hospital mortality [55]. The importance of appropriate treatment was recently confirmed in a study of *P. aeruginosa* bloodstream infections; although neither bloodstream infections with metallo-β-lactamase producing *P. aeruginosa* nor bloodstream infections with various resistance phenotypes of *P. aeruginosa* were independently associated with mortality or length of hospital stay [74]. Inadequate initial therapy that does not provide coverage for resistant *P. aeruginosa* is associated with poor clinical outcomes, longer hospital stays and higher costs [37, 75–78]. A prospective study examining patients with bloodstream infections due to *P. aeruginosa* found that inappropriate empirical antibiotic treatment was associated with a two-fold increased risk of mortality [79]. A retrospective study from a large tertiary hospital in the US also demonstrated that inappropriate initial antimicrobial treatment was an independent determinant of hospital mortality in patients with *P. aeruginosa* bloodstream infections and was associated with a doubling in the odds ratio for death [40]. The timing of initiation of therapy and site of infection were not considered within the meta-analysis due to a lack of comparable data, but should be an area for further research. Future analyses of the economic impact of resistance should also consider use of propensity scoring to reduce the impact of some these potential confounders [80].

This review and meta-analysis should be interpreted in light of a few limitations. Our inclusion criteria required that studies only consider nosocomial infection and were published in English [28–30]. Additionally, studies that were eligible for inclusion did not report on 30 day mortality. Finally, the literature is heterogeneous with respect to the definition of MDR and site of infection with *P. aeruginosa*. As a result, combined analysis may misrepresent the true picture within different diseases (e.g. ventilator-associated pneumonia versus complicated UTIs).

Confounding in observational studies reporting unadjusted data should be acknowledged. For example, patients with resistant *P. aeruginosa* represent a sicker population, who may have been treated with more antibiotics and may have had a longer length of stay. These factors may have influenced the outcome of in-hospital mortality rather than resistant organism, specifically. The choice of the control group (e.g. patients with infection with susceptible strains versus patients without any infection at all) may have also influenced the results of the meta-analyses. The review findings indicate that resistant *P. aeruginosa* can be a potential marker for increased in-hospital mortality. Analyzing the impact of resistance on length of stay and costs is difficult due to competing events of mortality and discharge or time dependent bias, which were not appropriately addressed in most of the included studies. With *P. aeruginosa,* the situation is further complicated by the emergence of resistance during therapy [12].

From a societal and policy perspective, several significant stakeholders who have a major role to play in combating the burgeoning public health threat of antimicrobial resistance include: legislators, regulatory authorities, payers, pharmaceutical companies, hospital systems and physicians. Hospitals will need to initiate surveillance programs, adopt progressive policies, and embrace antibiotic stewardship as not just a means to cut costs but improve individual patient outcomes and provide societal benefit. Payers will have to adopt policies that support use of new, appropriate and/or effective antibiotics. Finally physicians will need to develop and implement appropriate infection control measures and generate evidence to support clinical decision-making and enable a tailored approach to treating infections.

In conclusion, the findings of this analysis underscore the substantial clinical and economic costs associated with resistant and, particularly, MDR *P. aeruginosa* in hospitalized patients*.* Decision-makers must prioritize implementation of best practices, treatment pathways and incentive systems to improve outcomes in this patient population. Novel and evolving treatment strategies, such as increasing heterogeneity of antibiotics prescribing, should be further explored as a valuable strategy for minimizing the spread of resistance in *P. aeruginosa* in hospitals [81]. Additionally, this analysis emphasized the need to ensure consistency in the definition of resistance to allow for additional comparative analyses with outcomes other than mortality in the future.

## Appendix 1. Search strategy terms

The initial search strategy utilized was as follows: pseudomonas[tiab] AND (cross infection [82] OR nosocomial[tiab] OR hospital-acquired[tiab] OR healthcare-acquired[tiab] OR hospital-associated[tiab] OR healthcare-associated[tiab] OR ventilator-associated[tiab] OR hospital[tiab] OR hospitals[tiab] OR hospitalized[tiab] OR intensive care or critical care) AND eng[la] AND (prevalence [82] OR prevalence [tiab] OR rate[tiab] OR risk factors [82] OR mortality [82] OR mortality[tiab] OR economics[mesh subheading] OR economic[tiab] OR risk[tiab] OR “statistics and numerical data”[mesh subheading] OR mortality[mesh subheading] OR patterns[tiab] OR epidemiology[mesh subheading] OR Surveillance[tiab] OR “risk factors” OR epidemiology OR outcome* OR cost[tiab]) AND (resistant OR resistance OR MDR[tiab] OR susceptib*[tiab] OR nonsusceptib*[tiab]) NOT (pediatric OR child[tiab] OR children[tiab] OR neonate*[tiab] OR neonatal[tiab] OR infant[tiab] OR infants[tiab] OR inhalation[tiab] OR inhaled[tiab] OR burn[tiab] OR transplant*[tiab] NOT (review [83] OR letter [83] OR case reports [83] OR meta-analysis [83] OR editorial [83] OR comment [83]).

## Electronic supplementary material

Additional file 1: **Risk of Bias using Newcastle -** **Ottawa Quality Assessment Scale (Cohort).** (PDF 331 KB)

Additional file 2: **Multivariable Factors and Subgroup Analyses.** **Table S1.** Description of multivariable adjusted factors. **Table S2.** Subgroup Analyses of Unadjusted Mortality. **Table S3.** Subgroup Analyses of Adjusted Mortality. (DOCX 72 KB)

## Abbreviations

CI: Confidence Interval
CPI: Consumer Price Index
ICU: Intensive Care Unit
IQR: Interquartile Range
LOS: Length of Stay
MBL: Metallo-β-Lactamase
MDR: Multidrug-resistant
*P. aeruginosa*: *Pseudomonas aeruginosa*
RR: Relative Risk
SD: Standard Deviation.
