# Supplementary material for: Clinical and economic consequences of hospital-acquired resistant and multidrug-resistant Pseudomonas aeruginosa infections: a systematic review and meta-analysis
Source: Antimicrob Resist Infect Control. 2014 Oct 20;3:32. doi: 10.1186/2047-2994-3-32 (PMC4219028; doi:10.1186/2047-2994-3-32)
Supplement: Supplementary file 1 — Additional file 1: Risk of Bias using Newcastle - Ottawa Quality Assessment Scale (Cohort). (PDF 331 KB) [file 13756_2014_597_MOESM1_ESM.pdf]

Appendix Table 2. Risk of Bias using **Newcastle - Ottawa Quality Assessment Scale (Cohort)**

**Study (Year)**	Representativeness of the exposed cohort (1 star allowed)	Selection of the non exposed cohort	Ascertainment of exposure	Demonstration that outcome of interest was not present at start of study	Comparability of cohorts on the basis of the design or analysis (2 stars allowed)	Ascertainment of outcome (1 star allowed)	Was follow-up long enough for outcomes to occur	Adequacy of follow up of cohorts	Overall ROB
Akhabue *et al.* (2011)	*	*	*	*	* (unmatched; adjusted analysis)	*	*	*	Medium
Brooklyn Antibiotic Resistance Task Force (2002)	*	*	*	*	* (matched; unadjusted analysis)	*	*	*	Medium
Cao *et al.* (2004)	*	*	*	Unclear	* (unmatched; adjusted analysis)	Not described	Not described	Not described	High
Eagye *et al.* (2009)	*	*	*	*	* (unmatched; adjusted analysis)	*	*	*	Medium
Evans *et al.* (2007)	*	*	*	*	* (unmatched; adjusted	*	*	*	Medium

**Study (Year)**	Representativeness of the exposed cohort (1 star allowed)	Selection of the non exposed cohort	Ascertainment of exposure	Demonstration that outcome of interest was not present at start of study	Comparability of cohorts on the basis of the design or analysis (2 stars allowed)	Ascertainment of outcome (1 star allowed)	Was follow-up long enough for outcomes to occur	Adequacy of follow up of cohorts	Overall ROB
					analysis)				
Furtado *et al.* (2009)	*	*	*	*	* (excessively matched for outcome; adjusted analysis)	*	*	*	Medium
Furtado *et al*. (2011) **P**	*	*	*	*	(no adjusted analysis or matching)	*	*	*	High
Gasink *et al.* (2006)	* (subjects included more than once (ie, if they had *P. aeruginosa* isolates >30 days apart)	*	*	*	* (unmatched; adjusted analysis)	*	*	*	Medium
Hirakata *et al*. (2003)	*	*	*	*	* (Matched. No adjusted analyses)	*	*	*	Medium
Kaminski *et al.* (2011)	*	*	*	*	* * (matched for prior ICU stay; adjusted	*	*	*	Low

**Study (Year)**	Representativeness of the exposed cohort (1 star allowed)	Selection of the non exposed cohort	Ascertainment of exposure	Demonstration that outcome of interest was not present at start of study	Comparability of cohorts on the basis of the design or analysis (2 stars allowed)	Ascertainment of outcome (1 star allowed)	Was follow-up long enough for outcomes to occur	Adequacy of follow up of cohorts	Overall ROB
					analysis)				
Lambert *et al.* (2011) **P**	*	*	*	*	* (unmatched; adjusted analysis)	*	*	*	Medium
Lautenbach *et al.* (2010)	*	*	*	*	* (unmatched; adjusted analysis)	*	*	*	Medium
Montero *et al.* (2009)	*	*	*	*	* (matched for 3 variables; issue: matched variable also used in adjusted analysis)	*	*	*	Medium
Morales *et al.* (2012)	*	*	*	*	* (unmatched; adjusted analysis)	*	*	*	Medium

**Study (Year)**	Representativeness of the exposed cohort (1 star allowed)	Selection of the non exposed cohort	Ascertainment of exposure	Demonstration that outcome of interest was not present at start of study	Comparability of cohorts on the basis of the design or analysis (2 stars allowed)	Ascertainment of outcome (1 star allowed)	Was follow-up long enough for outcomes to occur	Adequacy of follow up of cohorts	Overall ROB
Paramythiotou *et al.* (2004)	*	*	*	*	* (matched for 2 variables; issue: reported outcome was used in matched variable)	*	*	*	Medium
Peña *et al.* (2013)	*	*	*	*	* (unmatched; adjusted analysis)	*	*	*	Medium
Scheetz *et al.* (2006)	*	*	*	*	(No adjusted analyses; unmatched)	*	*	*	High
Söderström *et al.* (2009)	*	*	*	*	* (Well matched. No adjusted analyses)	*	*	*	Medium
Tam *et al.* (2009)	*	*	*	*	(No adjusted analyses; unmatched)	*	*	*	High

**Study (Year)**	Representativeness of the exposed cohort (1 star allowed)	Selection of the non exposed cohort	Ascertainment of exposure	Demonstration that outcome of interest was not present at start of study	Comparability of cohorts on the basis of the design or analysis (2 stars allowed)	Ascertainment of outcome (1 star allowed)	Was follow-up long enough for outcomes to occur	Adequacy of follow up of cohorts	Overall ROB
Tam *et al.* (2010)	*	*	*	*	* * (matched; adjusted analysis)	*	*	*	Low
Trouillet *et al.* (2002) P	*	*	*	*	* (unmatched; adjusted analysis)	*	*	*	Medium
Tumbarello *et al.* (2013)	*	*	*	*	* (unmatched; adjusted analysis)	*	*	*	Medium
Zavascki *et al.* (2006) P	*	*	*	*	* (unmatched; adjusted analysis; comparison by outcome)	*	*	*	Medium

P=prospective studies
