# Supplementary material for: Clinical and economic consequences of hospital-acquired resistant and multidrug-resistant Pseudomonas aeruginosa infections: a systematic review and meta-analysis
Source: Antimicrob Resist Infect Control. 2014 Oct 20;3:32. doi: 10.1186/2047-2994-3-32 (PMC4219028; doi:10.1186/2047-2994-3-32)
Supplement: Supplementary file 2 — Additional file 2: Multivariable Factors and Subgroup Analyses. Table S1. Description of multivariable adjusted factors. Table S2. Subgroup Analyses of Unadjusted Mortality. Table S3. Subgroup Analyses of Adjusted Mortality. (DOCX 72 KB) [file 13756_2014_597_MOESM2_ESM.docx]

Clinical and economic consequences of hospital-acquired resistant and multidrug-resistant pseudomonas aeruginosa infections: a systematic review and meta-analysis

Dilip Nathwani^1^
Email: dilip.nathwani@nhs.net

Gowri Raman^2^
Email: graman@tuftsmedicalcenter.org

Katherine Sulham^3^
Email: kate.sulham@gmail.com

Meghan Gavaghan^3*^
^*^ Corresponding author
Email: meghan.gavaghan@gfk.com

Vandana Menon^4^
Email: vandana.menon@cubist.com

^1^ Ninewells Hospital and Medical School, Dundee, Scotland DD19SY, UK

^2^ Tufts Medical Center for Evidence Synthesis, Institute for Clinical Research and Health Policy Studies, 800 Washington Street, Box 63, Boston, MA 02111, USA

^3^ GfK Market Access, LLC, 21 Cochituate Rd, Wayland, MA 01778, USA

^4^ Cubist Pharmaceuticals, 65 Hayden Ave, Lexington, MA 02421, USA

Abstract

Background

Increasing rates of resistant and multidrug-resistant (MDR) P. aeruginosa in hospitalized patients constitute a major public health threat. We present a systematic review of the clinical and economic impact of this resistant pathogen.

Methods

Studies indexed in MEDLINE and Cochrane databases between January 2000-February 2013, and reported all-cause mortality, length of stay, hospital costs, readmission, or recurrence in at least 20 hospitalized patients with laboratory confirmed resistant P. aeruginosa infection were included. We accepted individual study definitions of MDR, and assessed study methodological quality.

Results

The most common definition of MDR was resistance to more than one agent in three or more categories of antibiotics. Twenty-three studies (7,881 patients with susceptible P. aeruginosa, 1,653 with resistant P. aeruginosa, 559 with MDR P. aeruginosa, 387 non-infected patients without P. aeruginosa) were analyzed. A random effects model meta-analysis was feasible for the endpoint of all-cause in-hospital mortality. All-cause mortality was 34% (95% confidence interval (CI) 27% – 41%) in patients with any resistant P. aeruginosa compared to 22% (95% CI 14% – 29%) with susceptible P. aeruginosa. The meta-analysis demonstrated a > 2-fold increased risk of mortality with MDR P. aeruginosa (relative risk (RR) 2.34, 95% CI 1.53 – 3.57) and a 24% increased risk with resistant P. aeruginosa (RR 1.24, 95% CI 1.11 – 1.38), compared to susceptible P. aeruginosa. An adjusted meta-analysis of data from seven studies demonstrated a statistically non-significant increased risk of mortality in patients with any resistant P. aeruginosa (adjusted RR 1.24, 95% CI 0.98 – 1.57). All three studies that reported infection-related mortality found a statistically significantly increased risk in patients with MDR P. aeruginosa compared to those with susceptible P. aeruginosa. Across studies, hospital length of stay (LOS) was higher in patients with resistant and MDR P. aeruginosa infections, compared to susceptible P. aeruginosa and control patients. Limitations included heterogeneity in MDR definition, restriction to nosocomial infections, and potential confounding in analyses.

Conclusions

Hospitalized patients with resistant and MDR P. aeruginosa infections appear to have increased all-cause mortality and LOS. The negative clinical and economic impact of these pathogens warrants in-depth evaluation of optimal infection prevention and stewardship strategies.

Keywords

Pseudomonas aeruginosa, Resistance, All-cause mortality

Background

Pseudomonas aeruginosa is a frequent causative pathogen in healthcare associated infections [1]. P. aeruginosa is the most common Gram-negative pathogen causing nosocomial pneumonia in the United States, and it is frequently implicated in hospital-acquired urinary tract and bloodstream infections [2-4]. In a point prevalence study conducted in Western European ICUs, P. aeruginosa was one of the most common organisms, constituting nearly a third (29%) of all Gram-negative isolates, and was present in 17% of all positive cultures [5]. The Infectious Disease Society of America includes P. aeruginosa in its list of ‘ESKAPE’ pathogens that pose the greatest public health threat due to a combination of increasing prevalence and ineffectiveness of existing antibacterial agents [6].

Rates of antibiotic resistant Gram-negative infections continue to rise worldwide, and effective therapeutic options against these infections are severely limited [7-9]. Each year in Europe, approximately 400,000 patients with hospital-acquired infections present with a resistant strain [10]. Resistance is a particular problem with P. aeruginosa, because of the low permeability of its cell wall [11,12] and its ability to acquire and express multiple resistance mechanisms including porin deletions and overexpression of efflux pumps [13-17].

While the prevalence of P. aeruginosa in the last two decades has remained stable, the prevalence of resistant strains has increased dramatically (Table 1) [18-26]. Resistant P. aeruginosa infections are associated with high mortality, morbidity, and increased resource utilization and costs [27-33]. Further, the acquisition of resistance during anti-pseudomonal therapy among initially susceptible isolates and the emergence of MDR isolates make treatment even more challenging [34]. The high prevalence of resistance and resultant limited treatment options leads to inappropriate empiric therapy [24,35], which is associated with poor clinical and economic outcomes [36-42].

**Table 1** Summary of data on rates of resistant Pseudomonas aeruginosa

| Author (Year) | Country | Setting | Rates of Resistance | Ref. |
| --- | --- | --- | --- | --- |
| NNIS (2004) | USA | ICU | Imipenem resistance = 15% | [18] |
|  |  |  | Quinolone resistance = 9% |  |
|  |  |  | 3rd-generation cephalosporin resistance = 20% |  |
| Obritsch et al. (2004) | USA | ICU | MDR P. aeruginosa (defined as resistance to at least three of the following four drugs: imipenem, ceftazidime, ciprofloxacin, and tobramycin) increased from 4% in 1993 to 14% in 2002 | [19] |
| Morrow et al. (2013) | USA | Tertiary | Doripenem resistance = 11.4% | [20] |
|  |  |  | Imipenem resistance = 21.9% |  |
|  |  |  | Meropenem resistance = 15.4% |  |
|  |  |  | Levofloxacin resistance = 26.0% |  |
|  |  |  | Ceftazidime resistance = 15.2% |  |
|  |  |  | Tobramycin resistance = 10.1% |  |
|  |  |  | Piperacillin / tazobactam resistance = 14.7% |  |
| Souli et al. (2008), European Center for Disease Prevention and Control (2013) | Europe | Tertiary | Carbapenem resistance = > 25% | [21,22] |
| De Francesco et al. (2013) | Italy | Tertiary | MDR P. aeruginosa (defined as resistance to 5 commonly prescribed antibiotics) increased from 2.1% in 2007 to 4.1% in 2010 | [23] |
| Joo et al. (2011) | Korea | Tertiary | Ceftazidime resistance = 37% | [24] |
|  |  |  | Piperacillin resistance = 22% |  |
|  |  |  | Imipenem resistance = 23% |  |
|  |  |  | Fluoroquinolone resistance = 24% |  |
|  |  |  | Aminoglycoside resistance = 18% |  |
| Gales et al. (2001) | South America | Tertiary | MDR resistance = 8.2% | [25] |
| Raja et al. (2001) | Malaysia | Tertiary | MDR resistance = 6.9% | [26] |

Several studies have examined the impact of resistant Gram-negative bacilli generally and MDR P. aeruginosa specifically, but there has not been an in-depth, comparative analysis of the contemporary literature reporting on mortality, morbidity and costs associated with resistant versus susceptible infection. This report is a systematic review of the clinical and economic consequences of resistant and MDR P. aeruginosa compared to susceptible P. aeruginosa and control patients without P. aeruginosa infections. We also conducted a meta-analysis of all-cause mortality to quantify the impact of resistant and MDR P. aeruginosa on this clinical outcome.

Methods

The authors followed standard systematic review methods [43]. A systematic search was conducted in the Cochrane Library and MEDLINE. In addition, the authors manually reviewed citations from retrieved articles to ensure inclusion of all relevant literature. Appendix 1 lists the initial search strategy terms related to the pathogen (P. aeruginosa), mode of infection (nosocomial, hospital-acquired, healthcare-acquired, hospital-associated, healthcare-associated, and ventilator-associated), and outcomes (resource utilization including length of stay, antibiotic use/duration, procedures, inpatient costs, readmission, recurrence, and death).

Study inclusion criteria included: article published in English language; publication date between January 1, 2000 and February 28, 2013; sample size of at least 20 patients; and adult hospitalized population. Articles published before 2000 were not included to ensure that the analysis focused on contemporary literature that reflects current infection rates, resistance patterns, and clinical practice guidelines. Exclusion criteria were applied to identify special patient population subsets in which study results would not provide data applicable to the general population. Studies were not limited by the source of infection, and all infection types were included as long as resistance was present (see Table 2 for a summary of sources of infection). Unpublished gray literature was not included and no authors were directly contacted for unpublished data.

**Table 2** Description of studies included in systematic review

| Study (Year) | Study Design | Setting/ location | Source of Infection | Definition of Resistance / Controls | Study Population | Patients (n) | Ref. |
| --- | --- | --- | --- | --- | --- | --- | --- |
| Akhabue et al. (2011) | Retrospective Observational | Tertiary; USA | Genitourinary, respiratory wound, blood, tissue | Culture positive results for cefepime susceptible or resistant P. aeruginosa | Cefepime resistant P. aeruginosa | 213 | [61] |
|  |  |  |  |  | Susceptible P. aeruginosa | 2316 |  |
| Brooklyn Antibiotic Resistance Task Force (2002) | Retrospective Observational | Tertiary; USA | Genitourinary, respiratory, wound | Culture positive results for carbapenem susceptible or resistant P. aeruginosa | Susceptible P. aeruginosa | 10 | [60] |
|  |  |  |  |  | Resistant P. aeruginosa | 10 |  |
| Cao et al. (2004) | Retrospective Observational | Tertiary; China | Genitourinary, respiratory wound, blood, tissue | Culture positive results for susceptible or MDR P. aeruginosa (when the absence of susceptibility to three or more antibiotics: ceftazidime, cefepime, piperacillin, ciprofloxacin, gentamicin, and imipenem or Meropenem) | MDR P. aeruginosa | 44 | [57] |
|  |  |  |  |  | Susceptible P. aeruginosa | 68 |  |
| Eagye et al. (2009) | Retrospective observational | Tertiary; USA | Genitourinary, respiratory, wound | Culture positive results for meropenem susceptible or resistant P. aeruginosa; control patients were located in the same unit and on the same day as cases and without 105 DRG codes related to infection | Meropenem resistant P. aeruginosa | 58 | [53] |
|  |  |  |  |  | Susceptible P. aeruginosa | 125 |  |
|  |  |  |  |  | Control | 57 |  |
| Evans et al. (2007) | Retrospective observational | Tertiary; USA | Genitourinary, respiratory, wound | Culture positive results for susceptible or resistant P. aeruginosa; resistance defined as resistance to all the drugs in one or more of the following antibiotic classes: aminoglycosides, cephalosporins, carbapenems, and fluoroquinolones. | Susceptible P. aeruginosa | 73 | [71] |
|  |  |  |  |  | Resistant P. aeruginosa | 47 |  |
| Furtado et al. (2009) | Retrospective observational | ICU; Brazil | Genitourinary, respiratory, wound | Culture positive results for imipenem susceptible or resistant P. aeruginosa; control patients were hospitalized in the same ICU unit as the case participants and matched by time (within a 30-day interval), age (within a 10-year interval), and time at risk (control patients were imipenem susceptible) | Imipenem resistant P. aeruginosa | 63 | [62] |
|  |  |  |  |  | Control | 182 |  |
| Furtado et al. (2011) | Prospective Observational | Tertiary; Brazil | Respiratory | Culture positive results for imipenem susceptible or resistant P. aeruginosa; Isolates were screened for the presence of metallo-β-lactamases by using multiplex PCR. | São Paulo Metallo-β-lactamase (SPM-1) producing imipenem resistant P. aeruginosa | 5 | [68] |
|  |  |  |  |  | Non-SPM-1-producing susceptible P. aeruginosa | 24 |  |
| Gasink et al. (2006) | Retrospective Observational | Tertiary; USA | Respiratory, wound | Culture positive results for flouroquinolone susceptible or resistant P. aeruginosa | Flouroquinolone resistant P. aeruginosa | 320 | [64] |
|  |  |  |  |  | Susceptible P. aeruginosa | 527 |  |
| Hirakata et al. (2003) | Retrospective Observational | Tertiary; Japan | Genitourinary, respiratory, wound | Culture positive results for bla_IMP_-positive or negative P. aeruginosa; control subjects selected at random from among inpatients; control subjects samples were bla_IMP_-negative P. aeruginosa | bla_IMP_-positive P. aeruginosa | 69 | [69] |
|  |  |  |  |  | bla_IMP_-negative P. aeruginosa | 247 |  |
| Kaminski et al. (2011) | Nested case cohort | ICU; France | Respiratory | Culture positive results for Ureido/carboxypenicillin susceptible or resistant P. aeruginosa | Ureido/carboxypenicillin-resistant P. aeruginosa | 70 | [66] |
|  |  |  |  |  | Susceptible P. aeruginosa | 153 |  |
| Lambert et al. (2011) | Prospective observational | ICU; EU | Respiratory | Susceptibility testing in different centers was done according to local policies | Ceftazidime resistant P. aeruginosa | 366 | [31] |
|  |  |  |  |  | Susceptible P. aeruginosa | 1266 |  |
| Lautenbach et al. (2010) | Retrospective Observational | Tertiary; USA | Genitourinary, respiratory wound, blood, tissue | Culture positive results for imipenem susceptible or resistant P. aeruginosa | Imipenem resistant P. aeruginosa | 253 | [63] |
|  |  |  |  |  | Susceptible P. aeruginosa | 2289 |  |
| Montero et al. (2009) | Retrospective Observational | Tertiary; Spain | Respiratory | Culture positive results for P. aeruginosa; MDR when the absence of susceptibility to three or more antibiotic families (betalactams, quinolones, carbapenems, and aminoglycosides); control patients also had nosocomial infection (20% with P. aeruginosa), but were not multidrug-resistant | MDR P. aeruginosa | 50 | [58] |
|  |  |  |  |  | Control | 50 |  |
| Morales et al. (2012) | Retrospective observational | Tertiary; Spain | Genitourinary, respiratory, wound | Culture positive results for susceptible or resistant P. aeruginosa; MDR when strains were resistant to carbapenems, β-lactams, quinolones, tobramycin, and gentamicin | MDR P. aeruginosa | 134 | [49] |
|  |  |  |  |  | Resistant P. aeruginosa | 119 |  |
|  |  |  |  |  | Susceptible P. aeruginosa | 149 |  |
| Paramythiotou et al. (2004) | Retrospective Observational (matched) | ICU; France | Genitourinary, respiratory, wound | Culture positive results for susceptible or resistant P. aeruginosa; MDR were resistant to piperacillin, ceftazidime, imipenem, and ciprofloxacin; control patient was hospitalized in the same ICU as the corresponding case participant during the study period but whose microbiological cultures for P. aeruginosa showed no growth at any time during their ICU stay. Controls matched to cases for length of ICU stay. | MDR P. aeruginosa | 37 | [54] |
|  |  |  |  |  | Control | 34 |  |
| Peña et al. (2013) | Retrospective observational | Tertiary; Spain | Respiratory | MDR defined as strains non-susceptible to > =1 agent in > =3 anti-pseudomonal antimicrobial categories (carbapenems, β-lactams, quinolones, tobramycin, and gentamicin) | MDR P. aeruginosa | 27 | [56] |
|  |  |  |  |  | Susceptible P. aeruginosa | 56 |  |
| Scheetz et al. (2006) | Retrospective Observational | Tertiary; USA | Blood | Culture positive results for fluoroquinolone susceptible or resistant P. aeruginosa | Fluoroquinolone resistant P. aeruginosa | 79 | [65] |
|  |  |  |  |  | Fluoroquinolone susceptible P. aeruginosa | 136 |  |
| Söderström et al. (2009) | Retrospective Observational (matched) | Tertiary; Finland | Wound | Culture positive results for resistant P. aeruginosa to ciprofloxacin, tobramycin, and a combination of piperacillin and tazobactam; A control participant with negative MDR P. aeruginosa culture was matched to each study participants (a drug-sensitive strain of P. aeruginosa was cultured from 31 control participants (48%) | MDR P. aeruginosa | 64 | [59] |
|  |  |  |  |  | Control | 64 |  |
| Tam et al. (2009) | Retrospective observational | Tertiary; USA | Blood | Culture positive results for AmpC++ by ceftazidime susceptibility with and without clavulanic acid | AmpC++ P. aeruginosa | 21 | [72] |
|  |  |  |  |  | Wild-type P. aeruginosa | 33 |  |
| Tam et al. (2010) | Retrospective observational | Tertiary; USA | Blood | MDR was defined as culture positive results for resistance to three or more of the following four classes of agents: antipseudomonal carbapenems, antipseudomonal β-lactams (penicillins and cephalosporins), aminoglycosides, and fluoroquinolones | MDR P. aeruginosa | 25 | [13] |
|  |  |  |  |  | Susceptible P. aeruginosa | 84 |  |
| Trouillet et al. (2002) | Prospective observational | ICU; France | Respiratory | Culture positive results for piperacillin susceptible or resistant P. aeruginosa | Piperacillin resistant P. aeruginosa | 34 | [67] |
|  |  |  |  |  | Piperacillin susceptible P. aeruginosa | 101 |  |
| Tumbarello et al. (2013) | Retrospective Observational | ICU; Italy | Respiratory | Culture positive results for piperacillin susceptible or resistant P. aeruginosa; MDR if in vitro resistance to >1 antipseudomonal agent in 3 or more of the following categories: ß-lactam/ß-lactamase inhibitors, cephalosporins, carbapenems, quinolones and aminoglycosides | MDR P. aeruginosa | 42 | [55] |
|  |  |  |  |  | Non-MDR P. aeruginosa | 68 |  |
| Zavascki et al. (2006) | Prospective observational | Tertiary; Brazil | Blood, genitourinary, respiratory, wound | Culture positive results for metallo-β-lactamase (MBL) –carrying versus non-MBL- carrying P. aeruginosa | MBL-carrying P. aeruginosa | 86 | [70] |
|  |  |  |  |  | Non-MBL-carrying P. aeruginosa | 212 |  |

Two researchers with training in evidence-based methods extracted relevant data for analysis. The extracted data included study design; participant characteristics; follow-up period; method of assessing resistant P. aeruginosa; association between resistance status and outcome; potential confounding variables adjusted for; method of ascertaining outcome; and statistical analyses. A web-based, automated data platform (Doctor Evidence, Santa Monica, CA) further cross-calculated the data to identify any numerical discrepancies (i.e., mismatches of sub-data to main population data, data reported in percentiles conflicting with unit data and vice versa). A third evidence-based review produced the final digital data repository. The web-based platform assessed identified studies for inclusion. Meta-analysis was conducted using the random effects method of weighting data for pooling [44]. The results are reported as summary relative risk (RR).

We considered the following outcomes for inclusion in a meta-analysis: all-cause mortality in hospital, length of stay (hospital and ICU), hospital costs or charges, microbiological infection clearance and readmissions. The random effects meta-analyses assessed any potential differential impact of resistant and MDR pathogens on the outcomes of interest using unadjusted and adjusted data, when available. All presented p-values were obtained from analysis within included studies. The Cochrane Q Chi-square test was used to test for heterogeneity of results across studies and quantified with I^2^ [45]. In addition, we combined studies using logit transformation of individual proportions and calculated their confidence interval (CI) and then back transformed the combined value. For meta-analyses with at least 10 studies, we evaluated the potential for publication bias with funnel plots and Egger’s tests for small study effects [46]. We looked for differences across studies using stratified analyses to explain heterogeneity in association results. To assess study quality, we applied quality questions from the Newcastle-Ottawa Quality Assessment Scales for case–control and observational studies [47]. When feasible, sensitivity analyses were conducted by excluding studies of high risk of bias. All analyses were performed in Stata version 13 (StataCorp, College Station, Texas).

Studies that used hospital costs or charges as a defined primary or secondary endpoint were included in this analysis. Hospital costs and charges were inflated to 2012 US dollars using the Consumer Price Index (CPI) as reported by the Bureau of Labor Statistics [48]. In one study where the costs were reported in Euros [49], conversion to USD was performed according the exchange rate at the time of the original analysis.

This review was based on evaluation of data from published studies and was exempt from ethics committee approval. In addition, this review did not involve any direct research on patients, and no informed consent was required.

Results and discussion

Study characteristics

Our literature search identified 1,970 potentially relevant references (1,967 references and three full-text articles identified through manual searching), which contained terms from the search strategy listed (Appendix 1). Based on review of abstracts only, 73 full text articles met the necessary inclusion criteria previously mentioned. After review of full text, an additional 50 articles were excluded based on the same criteria (Figure 1).

**Figure 1** Literature review study flow diagram.

Table 2 summarizes the definitions of controls, susceptibility, resistance, and MDR used in the studies included in the systematic review. Of note, consistent with other reports, we found that the definition of resistance and MDR in P. aeruginosa infections was inconsistent [50-52]. Resistance was either based on a class of antimicrobials or to a specific agent. Similarly, the most common definition for MDR was laboratory-confirmed resistance to more than one agent in three or more of categories of antibiotics, though one study required evidence of resistance to five different agents [49].

Of the 23 studies that met inclusion criteria, two were prospective observational studies, 18 were retrospective (Table 2). Of the 20 studies reporting on hospitalized patients, six specifically studied ICU patients. The 23 studies represented a total of 10,570 patients, of which 7,881 had susceptible P. aeruginosa infections, 1,653 had resistant P. aeruginosa infections, 559 had MDR P. aeruginosa infections, and 387 were control patients defined as non-infected patients without P. aeruginosa infections. Of the 23 studies, 2 were rated as good quality (low risk of bias), 17 were of moderate quality (medium risk of bias) and 4 were of poor quality (high risk of bias) (Additional file 1). Most studies included a mixture of patients with genitourinary, respiratory, wound, and bloodstream as the source of infection.

The most common definition (43.5% of studies) for MDR was laboratory-confirmed resistance to more than one agent in three or more of categories of antibiotics [13,17,49,53-59]. Resistance (non-MDR) was defined as laboratory confirmed resistance to one particular antibiotic agent. Analysis of resistance included one study on carbapenem-resistant P. aeruginosa (n = 10) [60], one study on cefepime-resistant P. aeruginosa (n = 213) [61], one study on meropenem-resistant P. aeruginosa (n = 58) [53], two studies on imipenem-resistant P. aeruginosa (n = 316) [62,63], two studies on fluoroquinolone-resistant P. aeruginosa (n = 399) [64,65], one study on ceftazidime-resistant P. aeruginosa (n = 366) [31], one study on ureido/carboxypenicillin-resistant P. aeruginosa (n = 70) [66] and one study on piperacillin-resistant P. aeruginosa (n = 34) [67] and three studies on metallo-β-lactamase-carrying P. aeruginosa (MBL-PA)[68-70]. Three studies included resistant P. aeruginosa patients but did not define resistance specific to any one treatment (n = 187) [49,71,72].

Three studies reported long-term clinical outcomes in patients with MDR P. aeruginosa, but did not provide data regarding in-hospital outcomes and therefore, were excluded from meta-analysis [58,59,72].

Of the seven outcomes examined, in-hospital all-cause mortality was the only endpoint for which a meta-analysis was feasible; when only ICU mortality was reported, those data were included in the analysis of in-hospital mortality. For the other six outcomes, a meta-analysis was not possible due to insufficient data or heterogeneity of data. For the all-cause mortality analysis, we were unable to stratify by the type of infection owing to the small sample size.

Mortality outcomes

Twenty studies reported data on hospital all-cause mortality (Table 3). In-hospital all-cause mortality ranged from 25 to 60% in the MDR P. aeruginosa group, 15 to 59% in the resistant P. aeruginosa group, and 7 to 50% in the susceptible P. aeruginosa group. Mortality was 34% (95% confidence interval (CI) 27% – 41%) in patients with resistant and MDR P. aeruginosa compared to 22% (95% CI 14% – 29%) with susceptible P. aeruginosa. When comparing patients with resistant P. aeruginosa infections versus those with susceptible P. aeruginosa infections, resistance was associated with 24% higher risk of in-hospital all-cause mortality (11 studies, 9,082 participants unadjusted RR 1.24, 95% CI 1.11 – 1.38; I^2^ 24.6%). Comparing patients with MDR P. aeruginosa infections with patients with susceptible P. aeruginosa infections (5 studies, 697 participants), the MDR P. aeruginosa group had a greater than 2-fold increase in risk of in-hospital all-cause mortality (unadjusted RR 2.34, 95% CI 1.53 – 3.57; I^2^ 78.8%).

**Table 3** Studies describing in-hospital mortality in patients with resistant and multidrug-resistant Pseudomonas aeruginosa infections

| Setting | Author | Study groups (n) | In-Hospital Mortality | Reported P-value | Ref |
| --- | --- | --- | --- | --- | --- |
| Tertiary care center | Akhabue et al. (2011) | Cefepime resistant P. aeruginosa (213) | 20% | 0.007 | [61] |
|  |  | Susceptible P. aeruginosa (2316) | 13% |  |  |
|  | Brooklyn Antibiotic Resistance Task Force (2002) | Carbapenem resistant P. aeruginosa (10) | 20% | > 0.05 (NS) | [60] |
|  |  | Susceptible P. aeruginosa (10) | 10% |  |  |
|  | Cao et al. (2004) | MDR P. aeruginosa^d^ (44) | 55% | 0.05 | [57] |
|  |  | Susceptible P. aeruginosa (68) | 16% |  |  |
|  | Eagye et al. (2009) | Meropenem resistant P. aeruginosa (58) | 31% | 0.152^a^ | [53] |
|  |  | Meropenem susceptible P. aeruginosa (125) | 15% |  |  |
|  |  | Meropenem resistant P. aeruginosa (58) | 31% | 0.01^a^ |  |
|  |  | Control (57) | 9% |  |  |
|  | Evans et al. (2007) | Resistant P. aeruginosa^e^ (47) | 15% | 0.43 | [71] |
|  |  | Susceptible P. aeruginosa (73) | 21% |  |  |
|  | Furtado et al. (2011) | SPM-1-producing imipenem resistant P. aeruginosa (5) | 60% | 0.59 | [68] |
|  |  | Non-SPM-1-producing susceptible P. aeruginosa (24) | 75% |  |  |
|  | Gasink et al. (2006) | Fluoroquinolone resistant P. aeruginosa (320) | 24% | 0.004 | [64] |
|  |  | Fluoroquinolone susceptible P. aeruginosa (527) | 16% |  |  |
|  | Hirakata et al. (2003) | bla_IMP_-positive P. aeruginosa (69) | 30.4% | 0.41 | [69] |
|  |  | bla_IMP_-negative P. aeruginosa (247) | 25.5% |  |  |
|  | Lautenbach et al. (2010) | Imipenem resistant P. aeruginosa (253) | 17% | 0.01 | [63] |
|  |  | Imipenem susceptible P. aeruginosa (2289) | 13% |  |  |
|  | Morales et al. (2012) | MDR P. aeruginosa^f^ (134) | 25% | < 0.05 | [49] |
|  |  | Susceptible P. aeruginosa (149) | 13% |  |  |
|  |  | Resistant P. aeruginosa (119) | 22% | <0.05 |  |
|  |  | Susceptible P. aeruginosa (149) | 13% |  |  |
|  | Peña et al. (2013) | Non-MDR P. aeruginosa (27) | 55% | 0.33 | [56] |
|  |  | MDR P. aeruginosa^g^ (56) | 50% |  |  |
|  | Scheetz et al. (2006) | Fluoroquinolone resistant P. aeruginosa (79) | 32% | 0.731 | [65] |
|  |  | Fluoroquinolone susceptible P. aeruginosa (136) | 29% |  |  |
|  | Tam et al. (2010) | MDR P. aeruginosa^h^ (25) | 56% | 0.001 | [13] |
|  |  | Susceptible P. aeruginosa (84) | 17% |  |  |
|  | Zavascki et al. (2006) | MBL-carrying P. aeruginosa (86) | 51.2% | 0.003 | [70] |
|  |  | Non-MBL-carrying P. aeruginosa (212) | 32.1% |  |  |
| ICU | Lambert et al. (2011) | Ceftazidime resistant P. aeruginosa (362)^b^ | 43% | NR | [31] |
|  |  | Susceptible P. aeruginosa (1251)^b^ | 37% |  |  |
|  |  | Ceftazidime resistant P. aeruginosa (82)^c^ | 41% |  |  |
|  |  | Susceptible P. aeruginosa (280)^c^ | 39% |  |  |
|  | Furtado et al. (2009) | Imipenem resistant P. aeruginosa (63) | 49% | 0.02 | [62] |
|  |  | Control (182) | 34% |  |  |
|  | Kaminski et al. (2011) | Ureido/Carboxypenicillin resistant P. aeruginosa (70) | 43% | 0.56 | [66] |
|  |  | Ureido/Carboxypenicillin susceptible P. aeruginosa (153) | 44% |  |  |
|  | Paramythiotou et al. (2004) | MDR P. aeruginosa^i^ (34) | 47% | 0.8 | [54] |
|  |  | Control (34) | 50% |  |  |
|  | Trouillet et al. (2002) | Piperacillin resistant P. aeruginosa (34) | 59% | > 0.05 (NS) | [67] |
|  |  | Piperacillin susceptible P. aeruginosa (101) | 50% |  |  |
|  | Tumbarello et al. 2013 | MDR P. aeruginosa^j^ (42) | 60% | 0.01 | [55] |
|  |  | Susceptible P. aeruginosa (68) | 35% |  |  |

^a^ Adjusted for time at risk; ^b^ Patients with pneumonia; ^c^ Patients with bloodstream infections; ^d^ Absence of susceptibility to three or more antibiotics: ceftazidime, cefepime, piperacillin, ciprofloxacin, gentamicin, and imipenem or Meropenem; ^e^ Absence of susceptibility to all the drugs in one or more of the following antibiotic classes: aminoglycosides, cephalosporins, carbapenems, and fluoroquinolones; ^f^ Absence of susceptibility to carbapenems, β-lactams, quinolones, tobramycin, and gentamicin; ^g^ Absence of susceptibility to one or more agent in three or more anti-pseudomonal antimicrobial categories (carbapenems, β-lactams, quinolones, tobramycin, and gentamicin); ^h^ Absence of susceptibility to three or more of the following four classes of agents: antipseudomonal carbapenems, antipseudomonal β-lactams (penicillins and cephalosporins), aminoglycosides, and fluoroquinolones; ^i^ Absence of susceptibility to piperacillin, ceftazidime, imipenem, and ciprofloxacin; ^j^ Absence of susceptibility to one or more antipseudomonal agent in 3 or more of the following categories: ß-lactam/ß-lactamase inhibitors, cephalosporins, carbapenems, quinolones and aminoglycosides.

We conducted a meta-analysis of 18 studies containing 20 comparisons (10,422 participants) that reported in-hospital all-cause mortality in patients with any resistant P.aeruginosa versus susceptible P. aeruginosa infections. The two additional comparisons came from one study that contributed data for both resistant and MDR P.aeruginosa groups [49] and another study that contained two different infection-site comparisons [63]. Using data reported from 18 studies, the meta-analysis demonstrated an increased risk of mortality with any resistant P. aeruginosa (unadjusted (RR) 1.53, 95% CI 1.28 – 1.82, I^2^ 73.1%), compared to susceptible P. aeruginosa (Figure 2).

**Figure 2** A forest plot of unadjusted in-hospital all-cause mortality comparing resistant and susceptible P. aeruginosa. HAP = Health-acquired Pneumonia. HABI = Health-acquired Blood Infection.

Only seven studies reported data for an adjusted meta-analysis (Additional file 2 Table 1); adjusted data from these studies demonstrated an increased risk of mortality (adjusted RR 1.24, 95% CI 0.98 – 1.57, I^2^ 57.5%) that did not reach statistical significance probably due to the small sample size (Figure 3). We were unable to perform comparisons against control patients without P. aeruginosa infections due to a scarcity of studies reporting mortality end points in this group of patients [55,62]).

**Figure 3** A forest plot of adjusted in-hospital all-cause mortality comparing resistant and susceptible P. aeruginosa. HAP = Health-acquired Pneumonia. HABI = Health-acquired Blood Infection.

For both unadjusted and adjusted data, stratified meta-analyses by study-level characteristics identified studies with prospective follow-up or those conducted at multi-center or ICU as less heterogeneous (Additional file 2 Tables 2-3); however, excluding high risk of bias studies did not change the overall estimate or measures of consistency (I^2^).

Funnel plots of all studies reporting unadjusted mortality (Figure 4) indicate a potential for missing studies with inverse associations (RR <1.0). Since <10 studies reported adjusted mortality data, we did not assess publication bias for this analysis.

**Figure 4** Funnel plot with pseudo 95% confidence limits.

Across three studies that reported infection-related mortality, all found a statistically significantly increased risk in patients with MDR P. aeruginosa compared to those with susceptible P. aeruginosa (Table 4).

**Table 4** Studies describing in-hospital mortality in patients with resistant and multidrug-resistant Pseudomonas aeruginosa infections – cause of death identified specifically as Pseudomonas aeruginosa infection

| Setting | Author | Study groups (n) | Infection-related In-Hospital Mortality | P-value | Ref |
| --- | --- | --- | --- | --- | --- |
| Tertiary Care Center | Cao et al. (2004) | MDR P. aeruginosa^a^ (44) | 45.5% | <0.05 | [57] |
|  |  | Susceptible P. aeruginosa (68) | 7.4% |  |  |
|  | Hirakata et al. (2003) | bla_IMP_-positive P. aeruginosa (69) | 5.8% | 0.02 | [69] |
|  |  | bla_IMP_-negative P. aeruginosa (247) | 1.2% |  |  |
|  | Tam et al. (2010) | MDR P. aeruginosa^b^ (25) | 52% | <0.001 | [13] |
|  |  | Susceptible P. aeruginosa (84) | 9.5% |  |  |

^a^ Absence of susceptibility to three or more antibiotics: ceftazidime, cefepime, piperacillin, ciprofloxacin, gentamicin, and imipenem or Meropenem; ^b^ Absence of susceptibility to three or more of the following four classes of agents: antipseudomonal carbapenems, antipseudomonal β-lactams (penicillins and cephalosporins), aminoglycosides, and fluoroquinolones

Economic outcomes

Ten studies containing 12 comparisons reported hospital length of stay (Table 5). Definitions and measures for length of stay varied across eligible studies making direct comparisons challenging. The study by Paramythiotou et. al. was a case control study that matched MDR P. aeruginosa cases and controls (defined as patients without P. aeruginosa infections) on the basis of median length of stay in the ICU (45 versus 44 days). Therefore, we did not include these control patients in analyses of comparative ICU length of stay. Of note, this study reported hospital stay following ICU discharge of 11.1 days (standard deviation [SD] of 18.6) in the MDR P. aeruginosa group and 5.8 days (SD 10.4) in the control group (p = 0.28) [54].

**Table 5** Studies describing hospital length of stay in patients with resistant and multidrug-resistant Pseudomonas aeruginosa infections

| Author | Study groups (n) | Median (mean) Hospital Length of Stay (days) | Reported Significance (p-value or 95% CI) | Ref |
| --- | --- | --- | --- | --- |
|  |  | Main findings |  |  |
| Brooklyn Antibiotic Resistance Task Force (2002)^*^ | Carbapenem susceptible P. aeruginosa (10) | 20 (17) | 0.002 (0.001) | [60] |
|  | Carbapenem resistant P. aeruginosa (10) | 33.5 (36) |  |  |
| Eagye et al. (2009)^*^ | Meropenem resistant P. aeruginosa (58) | 30 | <0.001 | [53] |
|  | Meropenem susceptible P. aeruginosa (125) | 16 |  |  |
|  | Meropenem resistant P. aeruginosa (58) | 30 | <0.001 |  |
|  | Control (57) | 10 |  |  |
| Evans et al. (2007)^‡^ | Susceptible P. aeruginosa (73) | 20 | 0.03 | [71] |
|  | Resistant P. aeruginosa^e^ (47) | 26 |  |  |
| Furtado et al. (2009)^‡^ | Imipenem resistant P. aeruginosa (63) | 25 | 0.006 | [62] |
|  | Control (182) | 15 |  |  |
| Gasink et al. (2006)^*^ | Fluoroquinolone resistant P. aeruginosa (320) | 10 | 0.13 | [64] |
|  | Fluoroquinolone susceptible P. aeruginosa (527) | 9 |  |  |
| Kaminski et al. (2011)^†^ | Ureido/Carboxypenicillin resistant P. aeruginosa (70) | 50 | 0.24 | [66] |
|  | Ureido/Carboxypenicillin susceptible P. aeruginosa (153) | 47.5 |  |  |
| Lautenbach et al. (2010)^†^ | Imipenem resistant P. aeruginosa (253) | 16 | <0.001 | [63] |
|  | Imipenem susceptible P. aeruginosa (2289) | 9 |  |  |
| Morales et al. (2012)^‡^ | MDR P. aeruginosa^f^ (134) | 39 (45.7) | <0.001^a^ | [49] |
|  | Susceptible P. aeruginosa (149) | 20 (25.1) |  |  |
|  | Resistant P. aeruginosa (119) | 30 (39.0) | <0.001^a^ |  |
|  | Susceptible P. aeruginosa (149) | 20 (25.1) |  |  |
| Paramythiotou et al. (2004)^†^ | MDR P. aeruginosa^g^ (34) | 44.5 (57.0)^b,d^/11.1^c,d^ | 0.28/0.81 | [54] |
|  | Control (34) | 43.5 (46.9) ^b,d^/5.8 ^c,d^ |  |  |
| Tam et al. (2010)^†^ | MDR P. aeruginosa^h^ (25) | 26.4^c^ | 0.12 | [13] |
|  | Susceptible P. aeruginosa (84) | 16.5^c^ |  |  |

^*^ Length of stay reported after identification of infection with P. aeruginosa; ^†^ Length of stay reported as total length of stay; ^‡^ Length of stay not defined as total length of stay or length of stay after identification of infection with P. aeruginosa; ^a^ P-value reported for mean length of stay; ^b^ Length of ICU stay (ICU stay matched between cases and controls); ^c^ Length of stay in hospital following ICU discharge; ^d^ Study reported mean length of stay; ^e^ Absence of susceptibility to all the drugs in one or more of the following antibiotic classes: aminoglycosides, cephalosporins, carbapenems, and fluoroquinolones; ^f^ Absence of susceptibility to carbapenems, β-lactams, quinolones, tobramycin, and gentamicin; ^g^ Absence of susceptibility to piperacillin, ceftazidime, imipenem, and ciprofloxacin; ^h^ Absence of susceptibility to three or more of the following four classes of agents: antipseudomonal carbapenems, antipseudomonal β-lactams (penicillins and cephalosporins), aminoglycosides, and fluoroquinolones.

Two studies reported mean hospital length of stay among patients with MDR P. aeruginosa; one study reported total length of stay (46 days, SD 29) [49] and the other reported mean length of hospital stay associated with bacteremia (26.4 days; SD 28.3) [13]. Among patients with resistant P. aeruginosa, 8 studies reported median hospital length of stay in patients with bacteremia ranging from 10 to 50 days [13,49,53,54,60,62-64,66,71]; and 6 studies reported median hospital length of stay in susceptible P. aeruginosa infections ranging from 9 to 48 days [13,49,53,60,63,64,66,71]. Two studies reported median hospital length of stay in control patients (10 and 15 days, respectively) [53,54,62].

Three studies examined length of stay in the ICU (Table 6). One study reported mean ICU length of stay of 47 days in patients with MDR P. aeruginosa [54]. As described previously, the control group in the analysis by Paramythiotou et. al. [54] matched control patients with cases on the basis of ICU length of stay and therefore we did not include this control group. Two studies reported median ICU length of stay of 13 days (interquartile range 2–36) versus 6 days (interquartile range 0–16) in one study [66,71] and was similar in both groups in the other study (29 days) [66,71].

**Table 6** Studies describing intensive care unit length of stay in patients with resistant and multidrug-resistant Pseudomonas aeruginosa infections

| Author | Study groups (n) | Median ICU Length of Stay (days) | Reported Significance (p-value or 95% CI) | Ref |
| --- | --- | --- | --- | --- |
|  |  | Main findings |  |  |
| Evans et al. (2007) | Susceptible P. aeruginosa (73) | 6 | 0.02 | [71] |
|  | Resistant P. aeruginosa^c^ (47) | 13 |  |  |
| Kaminski et al. (2011) | Ureido/Carboxypenicillin resistant P. aeruginosa (70) | 29 | 0.37 | [66] |
|  | Ureido/Carboxypenicillin susceptible P. aeruginosa (153) | 28.5 |  |  |
| Paramythiotou et al. (2004) | MDR P. aeruginosa^d^ (34) | 44.5 (57.0)^a,b^ | 0.55 | [54] |
|  | Control (34) | 43.5 (46.9) ^a,b^ |  |  |

^a^ Length of ICU stay (ICU stay matched between cases and controls); ^b^ Study reported mean length of stay; ^c^ Absence of susceptibility to all the drugs in one or more of the following antibiotic classes: aminoglycosides, cephalosporins, carbapenems, and fluoroquinolones; ^d^ Absence of susceptibility to piperacillin, ceftazidime, imipenem, and ciprofloxacin.

Five studies reported outcomes related to hospital costs (Table 7). Four of five studies reported inpatient care costs. Of the four studies that reported inpatient care costs, two [49,63] reported only those costs incurred after microbiological infection confirmation. One study reported hospital charges incurred after microbiological infection confirmation [64]. Additionally, one study reported costs from a hospital in Spain [49] and therefore cannot be directly compared to the remaining three studies that represent US hospital setting costs. Studies also varied in the reporting of mean versus median costs. For the two US studies that reported median costs, costs were higher in the resistant P. aeruginosa groups (median = $99,672, interquartile range (IQR) = $43,714 - $187,260 [71]; median = $100,704, IQR = $27,710 - $183,125 [53]) compared to patients with susceptible P. aeruginosa infections (median = $69,502, IQR = $24,853 - $113,933 [71]; median = $32,594, IQR = $13,112 - $100,702 [53]) and control patients (median = $25,744, IQR = $17,456 - $40,616 [71]).

**Table 7** Studies describing hospital costs or charges in patients with resistant and multidrug-resistant Pseudomonas aeruginosa infections

| Author | Study groups (n) | Description of cost | Hospital Inpatient Care Costs or Charges | Reported P-value | Ref |
| --- | --- | --- | --- | --- | --- |
|  |  |  | Main findings |  |  |
| Eagye et al. (2009) | Meropenem resistant P. aeruginosa (58) | Median total cost (IQR) | $100,704 ($27,710 – $183,125) | < 0.001 | [53] |
|  | Meropenem susceptible P. aeruginosa (125) |  | $32,594 ($13,112 – $100,702) |  |  |
|  | Meropenem resistant P. aeruginosa (58) |  | $100,704 ($27,710 – $183,125) | < 0.001 |  |
|  | Control (57) |  | $25,744 ($17,456 – $40,616) |  |  |
| Evans et al. (2007) | Susceptible P. aeruginosa (73) | Median total cost (IQR) | $99,672 ($43,714 – $187,260 | 0.015 | [71] |
|  | Resistant P. aeruginosa^a^ (47) |  | $69,502 ($24,853 – $113,933) |  |  |
| Gasink et al. (2006) | Fluoroquinolone resistant P. aeruginosa (320) | Median total charges (IQR)^*^ | $62,325 ($22,129 - $188,979) | 0.008 | [64] |
|  | Fluoroquinolone susceptible P. aeruginosa (527) |  | $48,733 ($18,760 - $124,820) |  |  |
| Lautenbach et al. (2010) | Imipenem resistant P. aeruginosa (253) | Mean hospital cost after culture sampling (95% CI) | $286,417 ($234,326 – $338,510) | < 0.001 | [63] |
|  | Imipenem susceptible P. aeruginosa (2289) |  | $189,274 ($172,428 – $206,121) |  |  |
| Morales et al. (2012) | MDR P. aeruginosa^b^ (134) | Mean hospital cost after culture sampling (median) | $13,178 ($5,745) | < 0.001^†^ | [49] |
|  | Susceptible P. aeruginosa (149) |  | $4,258 ($2,420) |  |  |
|  | Resistant P. aeruginosa (119) |  | $10,662 ($5,248) | < 0.001^†^ |  |
|  | Susceptible P. aeruginosa (149) |  | $4,258 ($2,420) |  |  |

^*^ Hospital charges reported after identification of infection with P. aeruginosa; ^†^ P-value reported for mean length of stay; ^a^ Absence of susceptibility to all the drugs in one or more of the following antibiotic classes: aminoglycosides, cephalosporins, carbapenems, and fluoroquinolones; ^b^ Absence of susceptibility to carbapenems, β-lactams, quinolones, tobramycin, and gentamicin.

Other outcomes

Need for mechanical ventilation was studied as an index of patient outcomes in one case control study; patients with MDR P. aeruginosa required more days of mechanical ventilation than patients with susceptible P. aeruginosa (15 versus 11 days) [55]. One study reported persistence of microbiological infection of 75% and clinical persistence or recurrence of 38% in patients with MDR P. aeruginosa infection versus 61% and 39% respectively, in the non-MDR P. aeruginosa group [56]. As noted in the Methods section, meta-analyses were not performed for these outcomes due to insufficient data and heterogeneity of the literature.

Conclusions

This systematic review of the literature identified some strengths and weaknesses in relation to published data on the impact of P.aeruginosa infections on clinical and economic outcomes. The meta-analysis of studies examining the impact of resistance or MDR infection indicates a significant increase in hospital all-cause mortality compared to infections due to susceptible pathogens. Patients with resistant and MDR P. aeruginosa infections versus those with susceptible P. aeruginosa infections had a relative risk of 1.24 and 2.31, respectively.

In keeping with previous literature [27,28,32], our review suggests that MDR and resistant P. aeruginosa infections are associated with higher hospital and ICU length of stay as an outcome compared to susceptible P. aeruginosa infections; however, there was a scarcity of studies examining length of stay in patients with resistant P. aeruginosa infections compared to control patients without infection. Further research is needed into the economic impact of resistant P. aeruginosa infections and future studies must take into account regional and national differences in standards of care, and other critical patient level factors including community versus hospital acquired infection, duration of hospitalization prior to the infection, severity of illness, the timeliness and appropriateness of therapy, time at risk of infection, and site of infection [73].

Among reviewed studies, only one study highlighted inadequate initial antibiotic therapy as a significant risk factor for in-hospital mortality [55]. The importance of appropriate treatment was recently confirmed in a study of P. aeruginosa blood stream infections; although neither blood stream infections with metallo-β-lactamase producing P. aeruginosa nor blood stream infections with various resistance phenotypes of P. aeruginosa were independently associated with mortality or length of hospital stay [74]. Inadequate initial therapy that does not provide coverage for resistant P. aeruginosa is associated with poor clinical outcomes, longer hospital stays and higher costs [37,75-78]. A prospective study examining patients with bloodstream infections due to P. aeruginosa found that inappropriate empirical antibiotic treatment was associated with a two-fold increased risk of mortality [79]. A retrospective study from a large tertiary hospital in the US also demonstrated that inappropriate initial antimicrobial treatment was an independent determinant of hospital mortality in patients with P. aeruginosa bloodstream infections and was associated with a doubling in the odds ratio for death [40]. The timing of initiation of therapy and site of infection were not considered within the meta-analysis due to a lack of comparable data, but should be an area for further research. Future analyses of the economic impact of resistance should also consider use of propensity scoring to reduce the impact of some these potential confounders [80].

This review and meta-analysis should be interpreted in light of a few limitations. Our inclusion criteria required that studies only consider nosocomial infection and were published in English [28-30]. Additionally, studies that were eligible for inclusion did not report on 30 day mortality. Finally, the literature is heterogeneous with respect to the definition of MDR and site of infection with P. aeruginosa. As a result, combined analysis may misrepresent the true picture within different diseases (e.g. ventilator-associated pneumonia versus complicated UTIs).

Confounding in observational studies reporting unadjusted data should be acknowledged. For example, patients with resistant P. aeruginosa represent a sicker population, who may have been treated with more antibiotics and may have had a longer length of stay. These factors may have influenced the outcome of in-hospital mortality rather than resistant organism, specifically. The choice of the control group (e.g. patients with infection with susceptible strains versus patients without any infection at all) may have also influenced the results of the meta-analyses. The review findings indicate that resistant P. aeruginosa can be a potential marker for increased in-hospital mortality. Analyzing the impact of resistance on length of stay and costs is difficult due to competing events of mortality and discharge or time dependent bias, which were not appropriately addressed in most of the included studies. With P. aeruginosa, the situation is further complicated by the emergence of resistance during therapy [12].

From a societal and policy perspective, several significant stakeholders who have a major role to play in combating the burgeoning public health threat of antimicrobial resistance include: legislators, regulatory authorities, payers, pharmaceutical companies, hospital systems and physicians. Hospitals will need to initiate surveillance programs, adopt progressive policies, and embrace antibiotic stewardship as not just a means to cut costs but improve individual patient outcomes and provide societal benefit. Payers will have to adopt policies that support use of new, appropriate and/or effective antibiotics. Finally physicians will need to develop and implement appropriate infection control measures and generate evidence to support clinical decision-making and enable a tailored approach to treating infections.

In conclusion, the findings of this analysis underscore the substantial clinical and economic costs associated with resistant and, particularly, MDR P. aeruginosa in hospitalized patients. Decision-makers must prioritize implementation of best practices, treatment pathways and incentive systems to improve outcomes in this patient population. Novel and evolving treatment strategies, such as increasing heterogeneity of antibiotics prescribing, should be further explored as a valuable strategy for minimizing the spread of resistance in P. aeruginosa in hospitals [81]. Additionally, this analysis emphasized the need to ensure consistency in the definition of resistance to allow for additional comparative analyses with outcomes other than mortality in the future.

Abbreviations

CI, Confidence Interval; CPI, Consumer Price Index; ICU, Intensive Care Unit; IQR, Interquartile Range; LOS, Length of Stay; MBL, Metallo-β-Lactamase; MDR, Multidrug-resistant; P. aeruginosa, Pseudomonas aeruginosa; RR, Relative Risk; SD, Standard Deviation.

Competing interests

Funding for the completion of the systematic literature review and meta-analysis was provided via an unrestricted grant by Cubist Pharmaceuticals.

D. Nathwani, G. Raman, K. Sulham and M. Gavaghan are paid consultants to Cubist Pharmaceuticals.

V. Menon is an employee of Cubist Pharmaceuticals.

Authors’ contributions

DN participated in designing the systematic review. GR participated in designing the systematic review, performed the statistical analysis and participated in drafting the manuscript. KS participated in designing the systematic review, performed the statistical analysis and participated in drafting the manuscript. MG participated in designing the systematic review, performed the statistical analysis and participated in drafting the manuscript. VM conceived of the study, participated in designing the systematic review, performed the statistical analysis and participated in drafting the manuscript. All authors read and approved the final manuscript.

Appendix 1. Search strategy terms

The initial search strategy utilized was as follows: pseudomonas[tiab] AND (cross infection [82] OR nosocomial[tiab] OR hospital-acquired[tiab] OR healthcare-acquired[tiab] OR hospital-associated[tiab] OR healthcare-associated[tiab] OR ventilator-associated[tiab] OR hospital[tiab] OR hospitals[tiab] OR hospitalized[tiab] OR intensive care or critical care) AND eng[la] AND (prevalence [82] OR prevalence [tiab] OR rate[tiab] OR risk factors [82] OR mortality [82] OR mortality[tiab] OR economics[mesh subheading] OR economic[tiab] OR risk[tiab] OR “statistics and numerical data”[mesh subheading] OR mortality[mesh subheading] OR patterns[tiab] OR epidemiology[mesh subheading] OR Surveillance[tiab] OR “risk factors” OR epidemiology OR outcome* OR cost[tiab]) AND (resistant OR resistance OR MDR[tiab] OR susceptib*[tiab] OR nonsusceptib*[tiab]) NOT (pediatric OR child[tiab] OR children[tiab] OR neonate*[tiab] OR neonatal[tiab] OR infant[tiab] OR infants[tiab] OR inhalation[tiab] OR inhaled[tiab] OR burn[tiab] OR transplant*[tiab] NOT (review [83] OR letter [83] OR case reports [83] OR meta-analysis [83] OR editorial [83] OR comment [83]).

References

1. Chatzinikolaou I, Abi-Said D, Bodey GP, Rolston KV, Tarrand JJ, Samonis G: Recent experience with Pseudomonas aeruginosa bacteremia in patients with cancer: Retrospective analysis of 245 episodes. Arch Intern Med 2000, 160:501–509.

2. Gaynes R, Edwards JR: Overview of nosocomial infections caused by gram-negative bacilli. Clin Infect Dis 2005, 41:848–854.

3. Hidron AI, Edwards JR, Patel J, Horan TC, Sievert DM, Pollock DA, Fridkin SK: NHSN annual update: antimicrobial-resistant pathogens associated with healthcare-associated infections: annual summary of data reported to the National Healthcare Safety Network at the Centers for Disease Control and Prevention, 2006–2007. Infect Control Hosp Epidemiol 2008, 29:996–1011.

4. Streit JM, Jones RN, Sader HS, Fritsche TR: Assessment of pathogen occurrences and resistance profiles among infected patients in the intensive care unit: report from the SENTRY Antimicrobial Surveillance Program (North America, 2001). Int J Antimicrob Agents 2004, 24:111–118.

5. Vincent JL, Rello J, Marshall J, Silva E, Anzueto A, Martin CD, Moreno R, Lipman J, Gomersall C, Sakr Y, Reinhart K: International study of the prevalence and outcomes of infection in intensive care units. JAMA 2009, 302:2323–2329.

6. Boucher HW, Talbot GH, Bradley JS, Edwards JE, Gilbert D, Rice LB, Scheld M, Spellberg B, Bartlett J: Bad bugs, no drugs: no ESKAPE! An update from the Infectious Diseases Society of America. Clin Infect Dis 2009, 48:1–12.

7. Peleg AY, Hooper DC: Hospital-acquired infections due to gram-negative bacteria. N Engl J Med 2010, 362:1804–1813.

8. Livermore DM: Has the era of untreatable infections arrived? J Antimicrob Chemother 2009, 64(Suppl 1):i29–i36.

9. Zhanel GG, DeCorby M, Adam H, Mulvey MR, McCracken M, Lagace-Wiens P, Nichol KA, Wierzbowski A, Baudry PJ, Tailor F, Karlowsky J, Walkty A, Schweizer F, Johnson J, the Canadian Antimicrobial Resistance Alliance, Hoban Dl: Prevalence of antimicrobial-resistant pathogens in Canadian hospitals: results of the Canadian Ward Surveillance Study (CANWARD 2008). Antimicrob Agents Chemother 2010, 54:4684–4693.

10. Antimicrobial Resistance: [http://www.euro.who.int/en/health-topics/disease-prevention/antimicrobial-resistance/antimicrobial-resistance].

11. Lambert PA: Mechanisms of antibiotic resistance in Pseudomonas aeruginosa. J R Soc Med 2002, 95(Suppl 41):22–26.

12. Breidenstein EB, de la Fuente-Nunez C, Hancock RE: Pseudomonas aeruginosa: all roads lead to resistance. Trends Microbiol 2011, 19:419–426.

13. Tam VH, Rogers CA, Chang KT, Weston JS, Caeiro JP, Garey KW: Impact of multidrug-resistant Pseudomonas aeruginosa bacteremia on patient outcomes. Antimicrob Agents Chemother 2010, 54:3717–3722.

14. Livermore DM: Multiple mechanisms of antimicrobial resistance in Pseudomonas aeruginosa: our worst nightmare? Clin Infect Dis 2002, 34:634–640.

15. Ho J, Tambyah PA, Paterson DL: Multiresistant Gram-negative infections: a global perspective. Curr Opin Infect Dis 2010, 23:546–553.

16. Lister PD, Wolter DJ, Hanson ND: Antibacterial-resistant Pseudomonas aeruginosa: clinical impact and complex regulation of chromosomally encoded resistance mechanisms. Clin Microbiol Rev 2009, 22:582–610.

17. Tam VH, Chang KT, Abdelraouf K, Brioso CG, Ameka M, McCaskey LA, Weston JS, Caeiro JP, Garey KW: Prevalence, resistance mechanisms, and susceptibility of multidrug-resistant bloodstream isolates of Pseudomonas aeruginosa. Antimicrob Agents Chemother 2010, 54:1160–1164.

18. National Nosocomial Infections Surveillance System: National Nosocomial Infections Surveillance (NNIS) System Report, data summary from January 1992 through June 2004, issued October 2004. Am J Infect Control 2004, 32:470–485.

19. Obritsch MD, Fish DN, MacLaren R, Jung R: National surveillance of antimicrobial resistance in Pseudomonas aeruginosa isolates obtained from intensive care unit patients from 1993 to 2002. Antimicrob Agents Chemother 2004, 48:4606–4610.

20. Morrow BJ, Pillar CM, Deane J, Sahm DF, Lynch AS, Flamm RK, Peterson J, Davies TA: Activities of carbapenem and comparator agents against contemporary US Pseudomonas aeruginosa isolates from the CAPITAL surveillance program. Diagn Microbiol Infect Dis 2013, 75:412–416.

21. Souli M, Galani I, Giamarellou H: Emergence of extensively drug-resistant and pandrug-resistant Gram-negative bacilli in Europe. Euro Surveill 2008, 13(47). Pii: 19045

22. European Center for Disease Prevention and Control: Antimicrobial Resistance Surveillance in Europe. ; 2013.

23. De Francesco MA, Ravizzola G, Peroni L, Bonfanti C, Manca N: Prevalence of multidrug-resistant Acinetobacter baumannii and Pseudomonas aeruginosa in an Italian hospital. J Infect Public Health 2013, 6:179–185.

24. Joo EJ, Kang CI, Ha YE, Park SY, Kang SJ, Wi YM, Lee NY, Chung DR, Peck KR, Song JH: Impact of inappropriate empiric antimicrobial therapy on outcome in Pseudomonas aeruginosa bacteraemia: a stratified analysis according to sites of infection. Infection 2011, 39:309–318.

25. Gales AC, Jones RN, Turnidge J, Rennie R, Ramphal R: Characterization of Pseudomonas aeruginosa isolates: occurrence rates, antimicrobial susceptibility patterns, and molecular typing in the global SENTRY Antimicrobial Surveillance Program, 1997–1999. Clin Infect Dis 2001, 32(Suppl 2):S146–S155.

26. Raja NS, Singh NN: Antimicrobial susceptibility pattern of clinical isolates of Pseudomonas aeruginosa in a tertiary care hospital. J Microbiol Immunol Infect 2007, 40:45–49.

27. Carmeli Y, Troillet N, Karchmer AW, Samore MH: Health and economic outcomes of antibiotic resistance in Pseudomonas aeruginosa. Arch Intern Med 1999, 159:1127–1132.

28. Aloush V, Navon-Venezia S, Seigman-Igra Y, Cabili S, Carmeli Y: Multidrug-resistant Pseudomonas aeruginosa: risk factors and clinical impact. Antimicrob Agents Chemother 2006, 50:43–48.

29. Kang CI, Kim SH, Park WB, Lee KD, Kim HB, Kim EC, Oh MD, Choe KW: Risk factors for antimicrobial resistance and influence of resistance on mortality in patients with bloodstream infection caused by Pseudomonas aeruginosa. Microb Drug Resist 2005, 11:68–74.

30. Lautenbach E, Weiner MG, Nachamkin I, Bilker WB, Sheridan A, Fishman NO: Imipenem resistance among pseudomonas aeruginosa isolates: risk factors for infection and impact of resistance on clinical and economic outcomes. Infect Control Hosp Epidemiol 2006, 27:893–900.

31. Lambert ML, Suetens C, Savey A, Palomar M, Hiesmayr M, Morales I, Agodi A, Frank U, Mertens K, Schumacher M, Wolkewitz M: Clinical outcomes of health-care-associated infections and antimicrobial resistance in patients admitted to European intensive-care units: a cohort study. Lancet Infect Dis 2011, 11:30–38.

32. Hirsch EB, Tam VH: Impact of multidrug-resistant Pseudomonas aeruginosa infection on patient outcomes. Expert Rev Pharmacoecon Outcomes Res 2010, 10:441–451.

33. Tambyah PA, Knasinski V, Maki DG: The direct costs of nosocomial catheter-associated urinary tract infection in the era of managed care. Infect Control Hosp Epidemiol 2002, 23:27–31.

34. Harris AD, Smith D, Johnson JA, Bradham DD, Roghmann MC: Risk factors for imipenem-resistant Pseudomonas aeruginosa among hospitalized patients. Clin Infect Dis 2002, 34:340–345.

35. Dupont H, Montravers P, Gauzit R, Veber B, Pouriat JL, Martin C: Outcome of postoperative pneumonia in the Eole study. Intensive Care Med 2003, 29:179–188.

36. Zilberberg MD, Chen J, Mody SH, Ramsey AM, Shorr AF: Imipenem resistance of Pseudomonas in pneumonia: a systematic literature review. BMC Pulm Med 2010, 10:45.

37. Kumar A, Ellis P, Arabi Y, Roberts D, Light B, Parrillo JE, Dodek P, Wood G, Kumar A, Simon D, Peters C, Ahsan M, Chateau D, Cooperative Antimicrobial Therapy of Septic Shock Database Research Groupl: Initiation of inappropriate antimicrobial therapy results in a fivefold reduction of survival in human septic shock. Chest 2009, 136:1237–1248.

38. Kollef MH: Update on the appropriate use of meropenem for the treatment of serious bacterial infections. Clin Infect Dis 2008, 47(Suppl 1):S1–S2.

39. Kang CI, Kim SH, Kim HB, Park SW, Choe YJ, Oh MD, Kim EC, Choe KW: Pseudomonas aeruginosa bacteremia: risk factors for mortality and influence of delayed receipt of effective antimicrobial therapy on clinical outcome. Clin Infect Dis 2003, 37:745–751.

40. Micek ST, Lloyd AE, Ritchie DJ, Reichley RM, Fraser VJ, Kollef MH: Pseudomonas aeruginosa bloodstream infection: importance of appropriate initial antimicrobial treatment. Antimicrob Agents Chemother 2005, 49:1306–1311.

41. Osih RB, McGregor JC, Rich SE, Moore AC, Furuno JP, Perencevich EN, Harris AD: Impact of empiric antibiotic therapy on outcomes in patients with Pseudomonas aeruginosa bacteremia. Antimicrob Agents Chemother 2007, 51:839–844.

42. Pop-Vicas A, Tacconelli E, Gravenstein S, Lu B, D’Agata EM: Influx of multidrug-resistant, gram-negative bacteria in the hospital setting and the role of elderly patients with bacterial bloodstream infection. Infect Control Hosp Epidemiol 2009, 30:325–331.

43. Moher D, Liberati A, Tetzlaff J, Altman DG, PRISMA Group: Preferred reporting items for systematic reviews and meta analyses: the PRISMA statement. Ann Intern Med 2009, 151:264–269. W64.

44. Borenstein M, Hedges LV, Higgins JPT, Rothstein HR: Generality of the basic inverse-variance method. In Introduction to Meta-Analysis. Chichester, UK: John Wiley & Sons LTd; 2009.

45. Huedo-Medina TB, Sanchez-Meca J, Marin-Martinez F, Botella J: Assessing heterogeneity in meta-analysis: Q statistic or I2 index? Psychol Methods 2006, 11:193–206.

46. Egger M, Davey SG, Schneider M, Minder C: Bias in meta-analysis detected by a simple, graphical test. BMJ 1997, 315(7109):629–634.

47. Wells G, Shea B, O’Connell D, Peterson J, Welch V, Losos M, Tugwell P: The Newcastle-Ottawa Scale (NOS) for Assessing the Quality of Nonrandomised Studies in Meta-analyses. ; 2007.

48. Consumer Price Index: [http://www.bls.gov/cpi/].

49. Morales E, Cots F, Sala M, Comas M, Belvis F, Riu M, Salvado M, Grau S, Horcajada JP, Montero MM, Castells X: Hospital costs of nosocomial multi-drug resistant Pseudomonas aeruginosa acquisition. BMC Health Serv Res 2012, 12:122.

50. Falagas ME, Koletsi PK, Bliziotis IA: The diversity of definitions of multidrug-resistant (MDR) and pandrug-resistant (PDR) Acinetobacter baumannii and Pseudomonas aeruginosa. J Med Microbiol 2006, 55:1619–1629.

51. Giske CG, Monnet DL, Cars O, Carmeli Y: Clinical and economic impact of common multidrug-resistant gram-negative bacilli. Antimicrob Agents Chemother 2008, 52:813–821.

52. Magiorakos AP, Srinivasan A, Carey RB, Carmeli Y, Falagas ME, Giske CG, Harbarth S, Hindler JF, Kahlmeter G, Olsson-Liljequist B, Paterson D, Rice L, Stelling J, Struelens M, Vatapoulos A, Weber J, Monnet D: Multidrug-resistant, extensively drug-resistant and pandrug-resistant bacteria: an international expert proposal for interim standard definitions for acquired resistance. Clin Microbiol Infect 2012, 18:268–281.

53. Eagye KJ, Kuti JL, Nicolau DP: Risk factors and outcomes associated with isolation of meropenem high-level-resistant Pseudomonas aeruginosa. Infect Control Hosp Epidemiol 2009, 30:746–752.

54. Paramythiotou E, Lucet JC, Timsit JF, Vanjak D, Paugam-Burtz C, Trouillet JL, Belloc S, Kassis N, Karabinis A, Andremont A: Acquisition of multidrug-resistant Pseudomonas aeruginosa in patients in intensive care units: role of antibiotics with antipseudomonal activity. Clin Infect Dis 2004, 38:670–677.

55. Tumbarello M, De Pascale G, Trecarichi EM, Spanu T, Antonicelli F, Maviglia R, Pennisi MA, Bello G, Antonelli M: Clinical outcomes of Pseudomonas aeruginosa pneumonia in intensive care unit patients. Intensive Care Med 2013, 39:682–692.

56. Pena C, Gomez-Zorrilla S, Oriol I, Tubau F, Dominguez MA, Pujol M, Ariza J: Impact of multidrug resistance on Pseudomonas aeruginosa ventilator-associated pneumonia outcome: predictors of early and crude mortality. Eur J Clin Microbiol Infect Dis 2013, 32:413–420.

57. Cao B, Wang H, Sun H, Zhu Y, Chen M: Risk factors and clinical outcomes of nosocomial multi-drug resistant Pseudomonas aeruginosa infections. J Hosp Infect 2004, 57:112–118.

58. Montero M, Dominguez M, Orozco-Levi M, Salvado M, Knobel H: Mortality of COPD patients infected with multi-resistant Pseudomonas aeruginosa: a case and control study. Infection 2009, 37:16–19.

59. Soderstrom M, Vikatmaa P, Lepantalo M, Aho PS, Kolho E, Ikonen T: The consequences of an outbreak of multidrug-resistant Pseudomonas aeruginosa among patients treated for critical leg ischemia. J Vasc Surg 2009, 50:806–812.

60. The Brooklyn Antiobotic Resistance Task Force: The cost of antibiotic resistance: effect of resistance among Staphylococcus aureus, Klebsiella pneumoniae, Acinetobacter baumannii, and Pseudmonas aeruginosa on length of hospital stay. Infect Control Hosp Epidemiol 2002, 23:106–108.

61. Akhabue E, Synnestvedt M, Weiner MG, Bilker WB, Lautenbach E: Cefepime-resistant Pseudomonas aeruginosa. Emerg Infect Dis 2011, 17:1037–1043.

62. Furtado GH, Bergamasco MD, Menezes FG, Marques D, Silva A, Perdiz LB, Wey SB, Medeiros EA: Imipenem-resistant Pseudomonas aeruginosa infection at a medical-surgical intensive care unit: risk factors and mortality. J Crit Care 2009, 24:625. e629-614.

63. Lautenbach E, Synnestvedt M, Weiner MG, Bilker WB, Vo L, Schein J, Kim M: Imipenem resistance in Pseudomonas aeruginosa: emergence, epidemiology, and impact on clinical and economic outcomes. Infect Control Hosp Epidemiol 2010, 31:47–53.

64. Gasink LB, Fishman NO, Weiner MG, Nachamkin I, Bilker WB, Lautenbach E: Fluoroquinolone-resistant Pseudomonas aeruginosa: assessment of risk factors and clinical impact. Am J Med 2006, 119:526. e519-525.

65. Scheetz MH, Bolon MK, Scarsi KK, Fotis MA, Postelnick MJ: Lack of effect of fluoroquinolone resistance on mortality in subjects with Pseudomonas aeruginosa bacteraemia. J Infect 2006, 52:105–110.

66. Kaminski C, Timsit JF, Dubois Y, Zahar JR, Garrouste-Orgeas M, Vesin A, Azoulay E, Feger C, Dumenil AS, Adrie C, Cohen Y, Allaouchiche B, the OUTCOMEREA study group: Impact of ureido/carboxypenicillin resistance on the prognosis of ventilator-associated pneumonia due to Pseudomonas aeruginosa. Crit Care 2011, 15:R112.

67. Trouillet JL, Vuagnat A, Combes A, Kassis N, Chastre J, Gibert C: Pseudomonas aeruginosa ventilator-associated pneumonia: comparison of episodes due to piperacillin-resistant versus piperacillin-susceptible organisms. Clin Infect Dis 2002, 34:1047–1054.

68. Furtado GH, Gales AC, Perdiz LB, Santos AF, Medeiros EA: Prevalence and clinical outcomes of episodes of ventilator-associated pneumonia caused by SPM-1-producing and non-producing imipenem-resistant Pseudomonas aeruginosa. Rev Soc Bras Med Trop 2011, 44:604–606.

69. Hirakata Y, Yamaguchi T, Nakano M, Izumikawa K, Mine M, Aoki S, Kondoh A, Matsuda J, Hirayama M, Yanagihara K, Miyazaki Y, Tomono K, Yamada Y, Kamihira S, Kohno S: Clinical and bacteriological characteristics of IMP-type metallo-beta-lactamase-producing Pseudomonas aeruginosa. Clin Infect Dis 2003, 37:26–32.

70. Zavascki AP, Barth AL, Goncalves AL, Moro AL, Fernandes JF, Martins AF, Ramos F, Goldani LZ: The influence of metallo-beta-lactamase production on mortality in nosocomial Pseudomonas aeruginosa infections. J Antimicrob Chemother 2006, 58:387–392.

71. Evans HL, Lefrak SN, Lyman J, Smith RL, Chong TW, McElearney ST, Schulman AR, Hughes MG, Raymond DP, Pruett TL, Sawyer RG: Cost of Gram-negative resistance. Crit Care Med 2007, 35:89–95.

72. Tam VH, Chang KT, Schilling AN, LaRocco MT, Genty LO, Garey KW: Impact of AmpC overexpression on outcomes of patients with Pseudomonas aeruginosa bacteremia. Diagn Microbiol Infect Dis 2009, 63:279–285.

73. Cosgrove SE, Carmeli Y: The impact of antimicrobial resistance on health and economic outcomes. Clin Infect Dis 2003, 36:1433–1437.

74. Willmann M, Kuebart I, Marschal M, Schroppel K, Vogel W, Flesch I, Markert U, Autenrieth IB, Holzl F, Peter S: Effect of metallo-beta-lactamase production and multidrug resistance on clinical outcomes in patients with Pseudomonas aeruginosa bloodstream infection: a retrospective cohort study. BMC Infect Dis 2013, 13:515.

75. Kumar A, Roberts D, Wood KE, Light B, Parrillo JE, Sharma S, Suppes R, Feinstein D, Zanotti S, Taiberg L, Gurka D, Kumar A, Cheang M: Duration of hypotension before initiation of effective antimicrobial therapy is the critical determinant of survival in human septic shock. Crit Care Med 2006, 34:1589–1596.

76. Kang CI, Kim SH, Park WB, Lee KD, Kim HB, Kim EC, Oh MD, Choe KW: Bloodstream infections caused by antibiotic-resistant gram-negative bacilli: risk factors for mortality and impact of inappropriate initial antimicrobial therapy on outcome. Antimicrob Agents Chemother 2005, 49:760–766.

77. Fraser A, Paul M, Almanasreh N, Tacconelli E, Frank U, Cauda R, Borok S, Cohen M, Andreassen S, Nielsen AD, Leibovici L: Benefit of appropriate empirical antibiotic treatment: thirty-day mortality and duration of hospital stay. Am J Med 2006, 119:970–976.

78. Leibovici L, Shraga I, Drucker M, Konigsberger H, Samra Z, Pitlik SD: The benefit of appropriate empirical antibiotic treatment in patients with bloodstream infection. J Intern Med 1998, 244:379–386.

79. Morata L, Cobos-Trigueros N, Martinez JA, Soriano A, Almela M, Marco F, Sterzik H, Nunez R, Hernandez C, Mensa J: Influence of multidrug resistance and appropriate empirical therapy on the 30-day mortality rate of Pseudomonas aeruginosa bacteremia. Antimicrob Agents Chemother 2012, 56:4833–4837.

80. Roberts RR, Hota B, Ahmad I, Scott RD 2nd, Foster SD, Abbasi F, Schabowski S, Kampe LM, Ciavarella GG, Supino M, Naples J, Cordell R, Levy S, Weinstein R: Hospital and societal costs of antimicrobial-resistant infections in a Chicago teaching hospital: implications for antibiotic stewardship. Clin Infect Dis 2009, 49:1175–1184.

81. Pluss-Suard C, Pannatier A, Kronenberg A, Muhlemann K, Zanetti G: Impact of antibiotic use on carbapenem resistance in Pseudomonas aeruginosa: is there a role for antibiotic diversity? Antimicrob Agents Chemother 2013, 57:1709–1713.

82. Marwick C, Santiago VH, McCowan C, Broomhall J, Davey P: Community acquired infections in older patients admitted to hospital from care homes versus the community: cohort study of microbiology and outcomes. BMC Geriatr 2013, 13:12.

83. Shorr AF, Tabak YP, Gupta V, Johannes RS, Liu LZ, Kollef MH: Morbidity and cost burden of methicillin-resistant Staphylococcus aureus in early onset ventilator-associated pneumonia. Crit Care 2006, 10:R97.

Additional files

**Additional_file_1 as PDF**
**Additional file 1** Risk of Bias using Newcastle - Ottawa Quality Assessment Scale (Cohort).

**Additional_file_2 as PDF**
**Additional file 2**
